# A 15‐Year Case Study of the Structures and Outcomes in a Blended Learning Postgraduate Master Programme for Periodontology and Implant Therapy

**DOI:** 10.1111/eje.70091

**Published:** 2026-02-21

**Authors:** P. Ratka‐Krüger, D. Arslan, M. Isailov‐Schoechlin, J. P. Woelber

**Affiliations:** ^1^ Department of Operative Dentistry and Periodontology, Faculty of Medicine University of Freiburg Freiburg Germany

**Keywords:** blended learning, continuing professional development, implant therapy, master program, periodontology, postgraduate education

## Abstract

**Aim:**

The master's program in Periodontology and Implant Therapy at the University of Freiburg, Germany, is one of the first continuing professional development (CPD) programs in periodontology delivered through a blended learning format. With over 15 years of successful operation, this study aimed to identify key factors contributing to its establishment and long‐term success.

**Materials and Methods:**

Data were collected through demographic analysis, an alumni survey, interviews with key individuals, and a review of publications, master theses, and academic awards. A multiperspective qualitative approach was used, addressing sociodemographic, user experience, pedagogical, technical, and scientific aspects. Responses from surveys and interviews were analysed using qualitative content analysis (Mayring's method) and systematically categorised.

**Results:**

The study analysed data from 106 master graduates, conducted a survey among 30 alumni, held 3 interviews and reviewed 24 publications, 96 master theses and 4 awards. The analysis showed that the blended learning program supported access to CPD for diverse groups, including participants from a broad range of age groups, students from rural areas, individuals with family responsibilities, and full‐time or self‐employed professionals. Personalised support and flexible structure were highlighted as major success factors, allowing learners to balance education with work and family life. Scientific output was also notable, with 24 publications (median Journal Impact Factor: 2.85).

**Conclusion:**

The integration of online learning, coordinated personal support, and in‐person phases were instrumental in the long‐term, successful establishment of the master's program in periodontology. These findings underscore the importance of flexible educational models, potentially serving as a model for future curricular CPD initiatives in dentistry.

## 1 | Introduction

Periodontal diseases are among the most prevalent diseases worldwide [1], with periodontitis ranking as the sixth most common disease globally [2]. Severe forms affect approximately 7.4% [3] of the population and are associated with substantial oral health and functional impairments, including tooth loss, masticatory dysfunction, aesthetic compromise, and reduced quality of life [2]. Given this background, it is crucial for dental students to receive thorough education in periodontology. While undergraduate (UG) dental curricula introduce preventive and basic periodontal therapy, their representation in curricula and allocated clinical treatment time remains disproportionately low compared to the high prevalence of periodontal cases in professional practice [4]. Moreover, they often lack the depth required for advanced procedures such as surgical treatment, complex risk management, or implant therapy [5, 6]. Consequently, there is a clear need for structured postgraduate training, particularly for practitioners intending to manage complex periodontitis cases.

Within the European Union, periodontology is formally recognised as a dental specialty [7]. In this context, the European Federation of Periodontology (EFP) has established the “Quality standards for graduate programs in periodontology, periodontics and implant dentistry” [8]. These standards embrace the concept of lifelong learning, which, according to the European Commission, is vital for adapting to new technologies and evolving scientific evidence in medical fields [9]. Continuing Professional Development (CPD) programs build on this principle, offering advanced training beyond initial qualification and enabling practitioners to integrate new technologies and scientific advancements into clinical care [10, 11]. This is particularly important in dentistry, where clinical procedures such as periodontal‐ and implant therapy demand high precision and ongoing skill refinement.

Traditionally, postgraduate training in periodontology has been conducted in face‐to‐face settings, requiring in‐person participation alongside professional practice. However, the increasing demand for flexible learning formats has led to the rise of blended‐learning models, which combine online instruction with hands‐on clinical training. These models allow professionals to balance education with their clinical responsibilities, making postgraduate training more accessible. The COVID‐19 pandemic has further underscored the need for digital agility in education, highlighting the importance of robust learning infrastructure to ensure continuity during crises [12].

Research suggests that blended learning is particularly effective in CPD programs, including those focused on surgical treatments in periodontology [4]. The Consensus Report of the Second European Consensus Workshop on Education in Periodontology emphasises the need for further research into the effects of blended learning on student engagement, performance, and learning outcomes in UG, PG, and CPD programs [13]. While face‐to‐face teaching remains highly valued, well‐structured hybrid models, integrating digital learning with supervised clinical training, have shown potential to enhance skill acquisition and professional competency [13].

However, the successful implementation of blended‐learning programs requires more than just digital integration. Key factors such as continuous curricular adaptation, ongoing participant support, and access to trained educators and pedagogical resources are critical for maintaining educational quality [4]. To address the growing need for flexible postgraduate education, the Ministry of Science, Research, and Arts of Baden‐Württemberg has supported an online master's program. In this context, the Master's Program in Periodontology and Implant Therapy was established in 2006 in Freiburg, Germany, with initial funding from the Ministry.

The three‐year, part‐time program comprises 120 ECTS across twelve modules. Approximately 91% of the content is delivered online, with 9% allocated to on‐site training (27 days over three years) at the University Dental Clinic in Freiburg. Modules combine self‐study, teletutoring, group assignments, and virtual classroom sessions. Practical competencies are taught during on‐site units focusing on diagnostics, surgical procedures (on animal and human specimens), and supervised clinical work. The program concludes with a master's thesis and oral defence (Table 1).

**TABLE 1:** Workload of the Master's Program in Periodontology and Implant Therapy.

Module	Online phase	On‐site session	Workload
Fundamentals & Diagnostics	8 weeks	3 days	150 h/19 h per week
Applied Anatomy	8 weeks	2 days	150 h/19 h per week
Oral Microbiology, Pathogenesis, and Pharmacology	13 weeks	3 days	150 h/11.5 h per week
Prophylaxis and Treatment Concept	13 weeks	3 days	200 h/15 h per week
Surgical Periodontal Therapy Part I	13 weeks	6 days	175 h/13.5 h per week
Surgical Periodontal Therapy Part II	13 weeks	3 days	175 h/13.5 h per week
Aesthetics & Function	22 weeks	3 days	200 h/9 h per week
Implantology	22 weeks	3 days	200 h/9 h per week
Master Module	26 weeks	1 day (oral exam)	325 h/13 h per week

Since its inception, the program has demonstrated remarkable continuity and adaptability. With over 15 years of experience, it remains unique in Europe as a long‐standing, structured blended‐learning program in postgraduate periodontology education. While the importance of blended‐learning approaches in postgraduate periodontology training has been acknowledged, there remains a notable lack of long‐term evidence evaluating effectiveness of blended‐learning CPD programs. As previously established by the Consensus Report of the Second European Consensus Workshop on Education in Periodontology [13], further research is needed to assess the impact of blended learning on learning outcomes. This study aimed to identify the key factors contributing to the sustained success of the Master's Program in Periodontology and Implant Therapy at the University of Freiburg. By analysing its development, structure, and outcomes, the study seeks to inform future postgraduate program design and contribute to the broader discourse on digital transformation in dental education.

## 2 Materials and Methods

**FIGURE 1:**
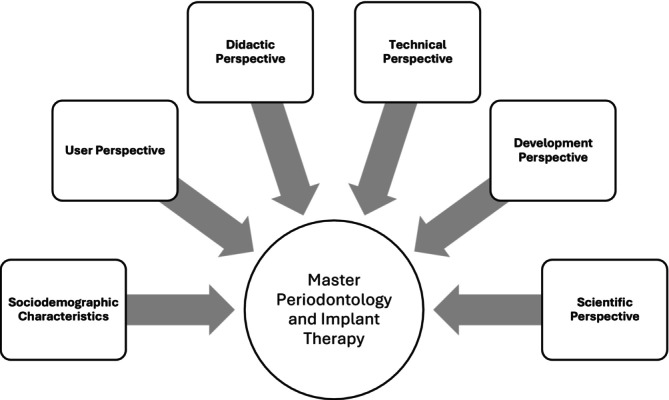
Domains of the multiperspective evaluation.

The study was designed as a multiperspective approach in mixed‐methods (quantitative analysis of 15‐years retrospective data, and qualitative interview analysis), specifically focusing on sociodemographic, user, pedagogical, technical, developmental, and scientific aspects (Figure 1).

### 2.1 Data Collection Methods

Data was collected through several approaches: a sociodemographic analysis of program alumni, an online survey among graduates, semi‐structured interviews with the management team of the Master's program, an examination of Master's theses, publications, and the research network stemming from the program, as well as a review of scientific awards received by the program and its faculty.

### 2.2 Sociodemographic Analysis

To provide context on the participant profile at the outset of the program, the age distribution of first‐year enrolments across 11 cohorts was analysed. Alumni data were analysed quantitatively with a focus on practice‐related characteristics, such as geographic distribution of workplaces. The analysis was based on anonymized internal lists provided by the program coordinator (reference year: 2019) and contextualised using external datasets from the Institute of German Dentists (IDZ) to enable benchmarking against broader national trends in dental practice.

### 2.3 Survey

To assess alumni perspectives on the Master's programme, an anonymous online survey was conducted using the platform UmfrageOnline.com (enuvo GmbH, Switzerland). Alumni were invited via email to participate. The questionnaire included two closed demographic questions on gender and age, as well as four open‐ended questions designed to elicit narrative feedback. The closed questions were internally developed based on established standards used in national health and education surveys (e.g., DESTATIS, IDZ) and reviewed by two researchers for clarity and relevance.

The open‐ended questions were as follows:

1. “What aspects of the Master's programme did you find particularly positive?”
2. “What aspects of the programme did you find negative or in need of improvement?”
3. “How did you experience the blended learning format?”
4. “Have there been any changes in your clinical practice as a result of completing the programme?”

These questions were developed by the research team to explore alumni reflections in depth, focusing on programme quality, didactic structure, and clinical impact. The use of open‐ended items, which cannot be answered with a simple ‘yes’ or ‘no’, allowed participants to share in‐depth insights based on their personal experiences. The survey layout followed the principles outlined by Steiner and Benesch [14], including an introductory information page that informed participants about data protection measures, anonymity, and data encryption. Responses to open‐ended questions were analysed using qualitative content analysis according to Mayring [15]. Each full statement provided by a respondent was treated as an individual Answer Unit (AU). A single participant could contribute multiple AUs per question. All AUs were inductively coded and grouped into thematic categories and subcategories to identify recurring patterns and insights.

### 2.4 Interviews

Semi‐structured interviews were conducted with key personnel involved in the Master's programme, including programme coordinators, faculty members, and technical staff. The aim was to gain insights into the developmental, scientific, technical, and pedagogical perspectives of the master's program. The interview guide was developed, piloted, and refined within the author group (PRK, DA, JW) in line with established methodological standards for semi‐structured interviewing and qualitative content analysis. The interviewer prepared and piloted an interview guide in advance and familiarised herself with recommended techniques through relevant literature (e.g., Mayring [15], Steiner and Benesch [14]). In a semi‐structured format, the interviewer prepared a set of predefined questions; the sequence in which they are posed remains flexible. This approach allows respondents to provide comprehensive, unrestricted responses, enabling an in‐depth exploration of complex topics while maintaining consistency in data collection. Interviews with key individuals, such as program coordinators and faculty members, were conducted to gather insights into the development, scientific, technical, and pedagogical perspectives of the master's program in Periodontology. The interviews were manually transcribed for analysis.

### 2.5 Review of Academic Achievements and Awards

The study included a review of master's theses, publications, and academic awards associated with the master's program. Master's theses were evaluated based on their subject matter. Publications were evaluated based on their academic impact, with a specific focus on their Journal Impact Factors (JIFs), as obtained from the Web of Science database (Clarivate, United Kingdom). The analysis aimed to assess the relevance, scholarly contribution, and overall influence of these works within their respective fields.

### 2.6 Evaluation

**FIGURE 2:**
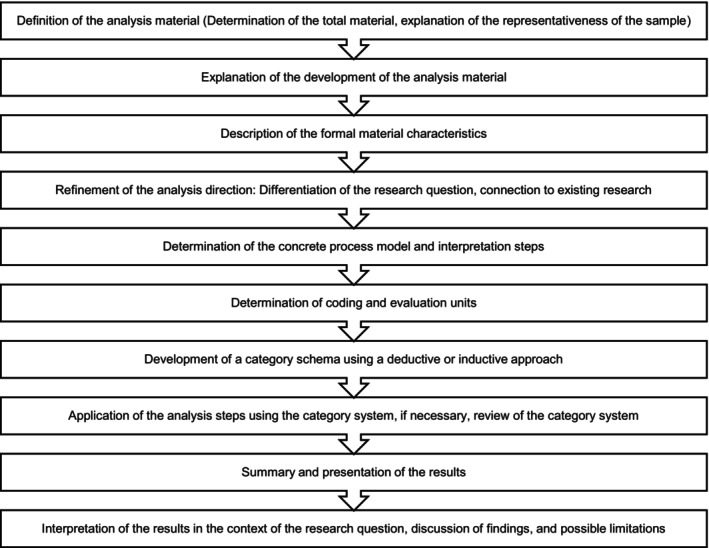
Content‐analytical process model of qualitative content analysis and its foundations and techniques [15].

Interview and survey results in this study were analysed using Mayring's qualitative content analysis [15] (Figure 2), which involved systematic category formation and the evaluation of responses to address the multifaceted aspects of the program. Data were coded and categorised into main and subcategories. These categories were defined and supported by anchor examples from the text. Each complete response was considered an answer unit (AU). The AUs per question can therefore be higher than the number of survey participants.

## 3 | Results

Interviews were conducted with three people involved in the program's administration: the program director, the coordinator, and the media technology coordinator. The study assessed sociodemographic data from 106 graduates of the master's program. The alumni survey, distributed to 96 participants, yielded 30 responses. Ninety‐six master's theses were systematically analysed. Additionally, a literature review was undertaken, covering 24 publications that resulted from the master's degree program, and four academic awards associated with the program and its faculty were reviewed (Table 2).

**TABLE 2:** Results of the collected data.

Data collection within the framework of multi‐perspective case representation			
Perspective	Target group	Data collection method	*n*
Development Perspective Scientific Perspective	Project Coordinator	Interviews	1
Technical Didactic Perspective	Technical Coordinator		1
Development Perspective Didactic Perspective	Program Director		1
User Perspective	Master's Graduates	Electronic/Written Survey	30
Scientific Perspective	Master's Graduates	List of all Completed Master's Theses	96
	Master's Graduates	Literature Review of Published Works	24

### 3.1 Interview Results

Interviews were conducted with the program director, the academic coordinator, and the media technology coordinator. The interviews yielded insights into the program's organisational, pedagogical, and technical development, as well as key implementation challenges. The findings are presented chronologically, reflecting the program's evolution over time.

The program was launched in 2006 with initial funding from the Ministry of Science, Research, and Arts of Baden‐Württemberg. The program's inception involved the establishment of a coordination team and the development of educational content and a didactic concept.

In 2007, the first student cohort commenced following successful accreditation by ACQUIN (Accreditation, Certification and Quality Assurance Institute), making it the first officially accredited online postgraduate program in periodontology in Germany.

In 2010, the program underwent major structural updates. These included a change in program name, the migration to the ILIAS learning management system (ILIAS open source e‐Learning e.V., Germany), and the implementation of an independent server system. These developments addressed growing technical demands and supported the expansion of teaching modules.

**FIGURE 3:**
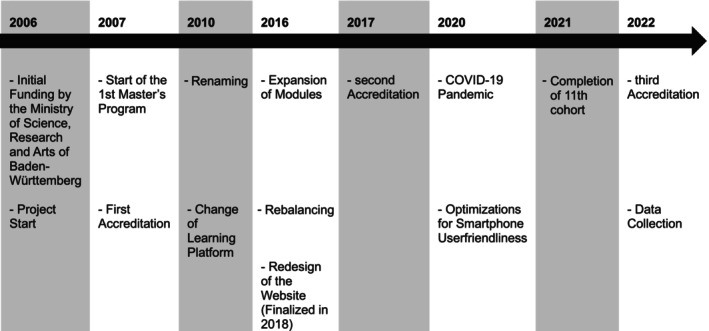
Timeline, development perspective on the master's program.

The second reaccreditation took place in 2013, again without conditions. In 2020, the COVID‐19 pandemic tested the digital infrastructure. The program responded by accelerating technical improvements, such as enhanced mobile compatibility and a more user‐friendly interface. These changes were based on results from a user survey, which had identified areas for technical and structural enhancement. The didactic framework of the program has continuously evolved, integrating new educational technologies and methodologies to optimise blended learning delivery. A third reaccreditation followed in December 2022, again without conditions (Figure 3).

### 3.2 Demographics and Geographic Distribution

**FIGURE 4:**
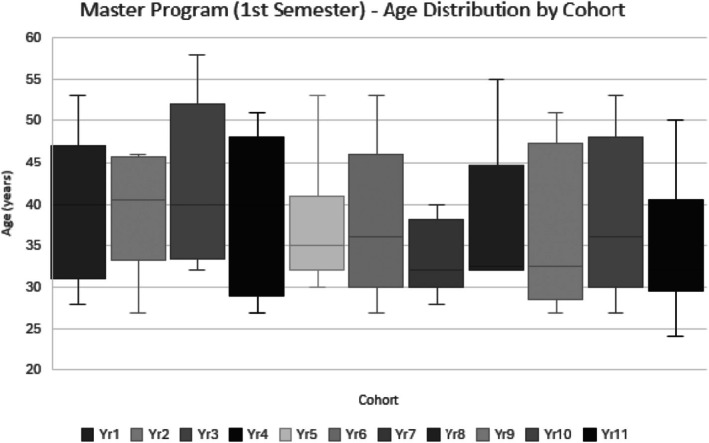
Age at enrolment by cohort (Yr1–Yr11). Box shows median and interquartile range; whiskers indicate minimum and maximum; points denote outliers where present.

The age distribution of first‐year enrolments across 11 cohorts showed a mean ± SD of 38.16 ± 8.23 years and a median of 36 years. Cohort‐wise age distributions at enrolment are shown in Figure 4 as box‐and‐whisker plots year 1–year 11 (Yr1–Yr11), illustrating variability within and between cohorts (Figure 4).

**FIGURE 5:**
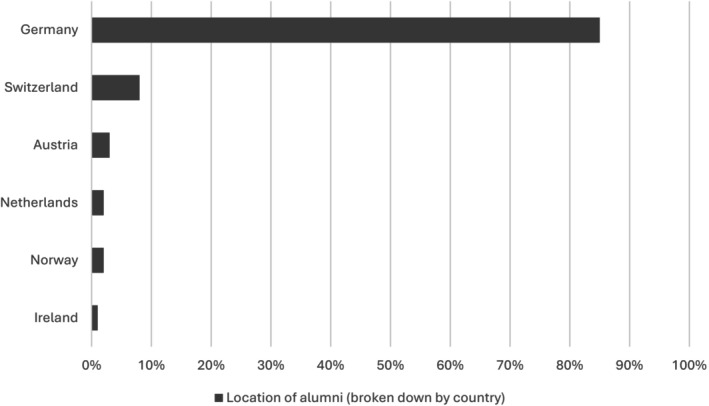
Location of alumni (broken down by country, in percent).

With the completion of its 11th cohort, the master's program in Periodontology and Implant Therapy had 106 alumni. The gender distribution among these graduates was 53% (*n* = 56) male and 47% (*n* = 50) female. While most (85%, *n* = 90) students were based in Germany, students were also located in Switzerland (8%, *n* = 8), Austria (3%, *n* = 3), the Netherlands (2%, *n* = 2), Norway (2%, *n* = 2) and Ireland (1%, *n* = 1) (Figure 5; Table S8, Supporting Information).

**FIGURE 6:**
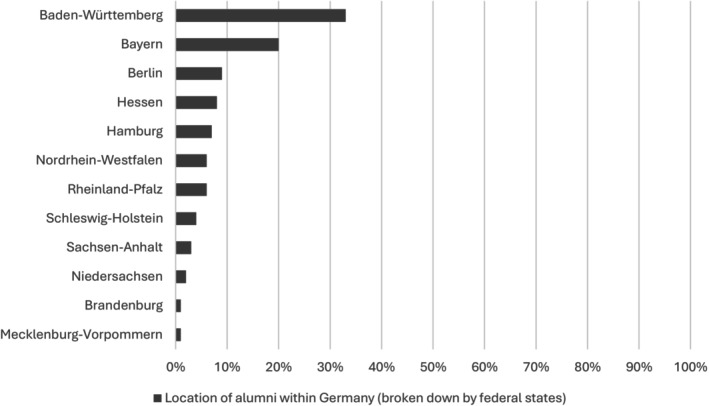
Location of alumni within Germany (broken down by federal states, in percent).

Within Germany, participants were primarily from the state of Baden‐Württemberg 33% (*n* = 30), where the university is located. Nevertheless, participants in the program were also from various other federal states across Germany, including Bayern 20% (*n* = 18), Berlin 9% (*n* = 8), Hessen 8% (*n* = 7), Hamburg 7% (*n* = 6), Nordrhein‐Westfalen 6% (*n* = 5), Rheinland‐Pfalz 6% (*n* = 5), Schleswig‐Holstein 4% (*n* = 4), Sachsen‐Anhalt 3% (*n* = 3), Niedersachsen 2% (*n* = 2), Brandenburg 1% (*n* = 1), and Mecklenburg‐Vorpommern 1% (*n* = 1) (Figure 6; Table S8, Supporting Information).

**FIGURE 7:**
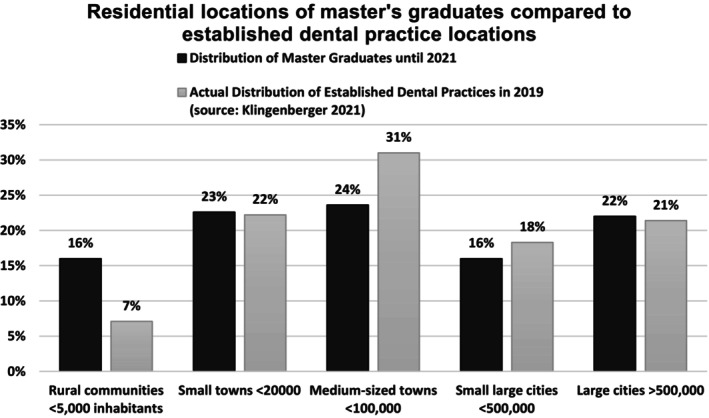
Comparison of master's graduates 2021 and established dental practices 2019.

To contextualise geographic patterns, alumni residential locations (2021) were compared with the distribution of established dental practices in Germany, as reported by the Institute of German Dentists (IDZ) [16] in 2019. Results revealed notable discrepancies: graduates from rural areas (population < 5000) were overrepresented (16%, *n* = 17), while those from medium‐sized towns (population < 100 000) were underrepresented (24%, *n* = 25). Graduates from small towns and large cities were proportionally represented in line with national dental practice distributions (Figure 7).

### 3.3 Survey Results: User Experience

**FIGURE 8:**
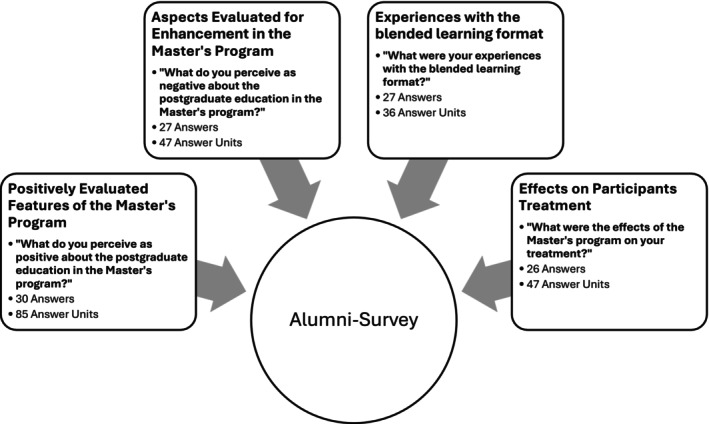
Questions of the Alumni Survey, gathered Answer and Answer Units.

To explore the user perspective, an anonymous online survey was distributed to 96 alumni via email. A total of 38 individuals responded, resulting in a response rate of 40%. Only fully completed surveys were included in the analysis, yielding 30 valid responses, corresponding to 31% of the total graduate population at the time of the survey. Participants ranged in age from 27 to 62 years. The gender distribution among respondents was 53% female (*n* = 16) and 47% male (*n* = 14). The survey examined participants' experiences with the Master's Program across four areas: Positively evaluated aspects of the program (85 answer units [AUs] from 30 respondents), areas identified for improvement (47 AUs from 27 respondents), experiences with the blended‐learning format (36 AUs from 27 respondents), and perceived effects on clinical treatment approaches (47 AUs from 26 respondents) (Tables 3, 4, 5, 6; original results of the alumni survey are provided in S1–S4, Supporting Information) (Figure 8).

**TABLE 3:** Positively Evaluated Features of the Master's Program divided into main and subcategories, (*n* = absolute number of AUs, % = proportion of responses rounded to the nearest whole number, based on a total of 85 AUs).

Positively Evaluated Features of the Master's Program		
Main category	Subcategory	Example for an AU [I consider as positive, …]
(I) Concept (42%, *n* = 36)	(I a) Blended learning	*“The time‐efficient combination of online and in‐person teaching for postgraduate students.”* (*1:48*, Supporting Information)
	(25%, *n* = 21)	*“[that the] regular work routine was possible [and the] reduced effort [since] travel was only necessary for in‐person events.”* (*1:52*, Supporting Information)
	(I b) Face‐to‐face phases (7%, *n* = 6)	*“The training is very comprehensive and diverse. In particular, the in‐person sessions have remained a great memory and were highly productive.”* (*1:64*, Supporting Information)
	(I c) Virtual classroom (5%, *n* = 4)	*“[The] support from the virtual classroom with assignments ensures time discipline and keeps human interaction with fellow students and lecturers alive.”* (*1:73*, Supporting Information)
	(I d) Program structure (4%, *n* = 3)	*“[The] well‐founded training with a consistent and coherent structure.”* (*1:74*, Supporting Information)
	(I e) Postgraduate program (2%, *n* = 2)	*“The well‐coordinated postgraduate program.”* (*1:77*, Supporting Information)
(II) Content (25%, *n* = 21)	(II a) General subject matter (15%, *n* = 13)	*“[The course content] led to a better understanding of periodontology, a more objective view of dentistry in general, and a deeper comprehension of biology.”* (*1:29*, Supporting Information)
	(II b) Applicability (5%, *n* = 4)	*“[The] comprehensive periodontal concept was convincing and is still fully integrated into daily practice.”* (*1:36*, Supporting Information)
	(II c) Surgery (5%, *n* = 4)	*“[The] learning of surgical techniques.”* (*1:40*, Supporting Information)
(III) Teaching Staff (25%, *n* = 21)	(III a) Supervisory ratio	*“[The] good supervisory ratio.”* (*1:1*, Supporting Information)
	(8%, *n* = 7)	*“The very pleasant atmosphere [and the] reasonably sized groups.”* (*1:3*, Supporting Information)
	(III b) Lecturers (7%, *n* = 6)	*“[The] many different lecturers from various universities.”* (*1:13*, Supporting Information)
	(III c) Team (4%, *n* = 3)	*“[The] highly qualified teaching staff*. “(*1:21*, Supporting Information)
	(III d) Faculty Leader (4%, *n* = 3)	*“[The] excellent lead professor. “*(*1:16*, Supporting Information)
	(III e) Teletutors (2%, *n* = 2)	*“[The] good availability and competence of the tele‐tutors.“*(*1:18*, Supporting Information)
(IV) Networking (8%, *n* = 7)	(IV) Peer exchange (8%, *n* = 7)	*„[The] interaction with colleagues who have a strong professional interest and enthusiasm for the field. “*(*1:83*, Supporting Information)

Data were collected through a defined category scheme developed and adapted based on the participants' responses. The analysis of answers to the first question, which addressed positively evaluated aspects of the program, identified four main thematic categories: the program's concept, the scientific content, the quality of the teaching staff, and networking opportunities (Table 4; original results of the alumni survey are provided in S1, Supporting Information).

**TABLE 4:** Aspects Evaluated for Enhancement in the Master's Program, divided into main and subcategories, (*n* = absolute number of AUs, % = proportion of responses rounded to the nearest whole number, based on a total of 47 AUs).

Aspects Evaluated for Enhancement in the Master's Program		
Main category	Subcategory	Example for an AU
(I) Content 40% (*n* = 19)	(I a) General Subject Matter 15% (*n* = 7)	*“It is unfortunate but understandable that access to literature databases was no longer available after completing the program. However, concerns that arose during the course were thoroughly addressed and discussed with lecturers and organisers, and adjustments were made accordingly.”* (*2:15*, Supporting Information)
	(I b) Surgery 11% (*n* = 5)	*“[The] surgery on patients is, on the one hand, a very well‐designed module; on the other hand, it would of course be desirable to perform an additional procedure, for example. However, it is clear that this would significantly increase the additional workload.”* (*2:4*, Supporting Information)
	(I c) Applicability 6% (*n* = 3)	*“Some presentations were highly scientific but lacked practical applicability for me.”* (*2:1*, Supporting Information)
	(I d) Reimbursement 4% (*n* = 2)	*“The discussion of economic aspects in periodontology was missing for me.”* (*2:17*, Supporting Information)
	(I e) Peri‐implantitis Therapy 4% (*n* = 2)	*“I was missing the peri‐implantitis therapy.”* (*2:18*, Supporting Information)
(II) Concept 40% (*n* = 19)	(II a) Face‐to‐face phases 19% (*n* = 9)	*“Primarily the long travel distance to Freiburg, although this is, of course, a purely subjective factor.”* (*2:31*, Supporting Information)
	(II b) High academic standards 13% (*n* = 6)	*“[The] academic rigour was high, and the workload for the master's thesis and patient cases was demanding.”* (*2:23*, *v*) *“Many assignments were difficult to manage alongside full‐time employment and childcare.”* (*2:24*, Supporting Information)
	(II c) Structure 6% (*n* = 3)	*“Since we were the first cohort and many participants were highly qualified, it became evident that some organisational adjustments were necessary. However, this was not entirely negative, as discussions and collegial exchanges were used to find solutions for everything.”* (*2:22*, Supporting Information)
	(II d) Virtual classroom 2% (*n* = 1)	*“The virtual classrooms were not initially effective enough to be of help to me.”* (*2:38*, Supporting Information)
(III) No Areas Identified for Enhancement 11% (*n* = 5)	(III a) No Areas Identified for Enhancement 11% (*n* = 5)	*“Strictly speaking, there is nothing negative.”* (*2:40*, Supporting Information)
(IV) Teaching Staff 4% (*n* = 2)	(IV a) Teaching Staff 4% (*n* = 2)	*“The only issue was that one or two lecturers were not up to date with their presentations or completely missed the mark in selecting exam questions.”* (*2:47*, Supporting Information)
(V) Networking 4% (*n* = 2)	(V a) Networking 4% (*n* = 2)	*“Complaining among fellow students regarding topics such as lunch, punctuality, and similar matters.”* (*2:44*, Supporting Information)

The blended‐learning structure of the postgraduate program was rated positively, particularly regarding its efficiency and flexibility in allowing participants to manage professional, academic, and personal commitments while minimising travel obligations. The limited requirement for in‐person attendance was frequently cited as a factor contributing to the program's accessibility, as participants associated it with a reduction in travel time and an optimised balance between study and work responsibilities. The combination of online and face‐to‐face teaching was also recognised as beneficial, as it was perceived to facilitate time‐efficient yet comprehensive learning experiences. Participants described the face‐to‐face sessions as highly productive, emphasising their contribution to the overall learning process. Concurrently, the virtual classroom was acknowledged for its role in promoting structured learning, time discipline, and continuous engagement with peers and faculty members. The academic content received strong evaluations, particularly regarding its scientific depth and clinical relevance. The curriculum was reported to enhance understanding of periodontology, provide a more objective perspective on dentistry, and deepen knowledge of biological principles. The comprehensive periodontal framework was repeatedly referenced as an effective educational component, with its integration into clinical practice viewed as an important outcome. Additionally, the surgical training component was frequently associated with direct applicability to clinical procedures. The quality of the teaching staff was a key aspect of the program's positive evaluation. Participants highlighted a favourable supervisory ratio and pleasant group sizes, both of which were perceived as contributing to a supportive and effective learning environment. The diverse range of lecturers was valued for ensuring broad coverage of key topics, while the faculty's expertise, the program's head professor, and the accessibility of tele‐tutors were frequently mentioned as factors that enhanced the overall educational experience. Networking opportunities were another positively assessed element. Participants emphasised the value of peer exchange and the opportunity to engage with colleagues who demonstrated a high level of professional commitment and interest in the field.

Although areas for improvement were evaluated less frequently in the second question, they were systematically categorised into comparable categories (Table 4; original results of the alumni survey are provided in S2, Supporting Information).

The responses highlighted key aspects of content, program structure, faculty, and networking. While the academic rigour and workload were recognised as demanding, they were seen as integral to the program's depth and quality. Managing the master's thesis and patient cases alongside professional and personal commitments as described as challenging but reflective of the high academic standards. The face‐to‐face sessions contributed significantly to the learning experience, though some participants noted that travel requirements posed logistical challenges. Regarding course content, participants valued its scientific depth but identified opportunities to strengthen practical applicability. Suggestions included expanding supervised surgical procedures, enhancing the integration of peri‐implantitis therapy, and providing more guidance on reimbursement policies to support clinical implementation. The program's structure was described as well‐organised, with faculty and peer discussions playing a key role in adapting to evolving needs. Participants recognised that continuous refinement is inherent in a dynamic academic environment. In assessing the impact on clinical practice, the program was credited with advancing treatment approaches, surgical skills, and professional confidence. Some participants noted that applying newly acquired techniques in daily practice could be complex due to external factors, highlighting the ongoing interplay between academic training and professional realities. Experiences with the blended learning format were predominantly positive (Table 5; original results of the alumni survey are provided in S3, Supporting Information).

**TABLE 5:** Experiences with the blended learning format, divided into main‐ and subcategories (*n* = absolute number of AUs, % = proportion of responses rounded to the nearest whole number, based on a total of 36 AUs).

Experiences with the blended learning format		
Main category	Subcategory	Example for an AU
(I) Positively Evaluated 86% (*n* = 31)	(I a) General Acknowledgment 25% (*n* = 9)	*“A very good approach to work‐integrated learning.”* (*3:9*, Supporting Information)
	(I b) Ratio of Online to In‐Person Teaching 17% (*n* = 6)	*“One can engage both online and in‐person to ask questions or deepen their understanding of the material. The balance between the two formats was well‐maintained.”* (*3:25*, Supporting Information)
	(I c) Gained Perspectives 14% (*n* = 5)	*“I had no prior experience with this. The Adobe platform was new to me, and I was so impressed that the Vorarlberg Dental Association adopted the Adobe concept for teaching and continuing education! This was particularly validated and proven effective during the COVID‐19 pandemic.”* (*3:27*, Supporting Information)
	(I d) Time Sovereignty 14% (*n* = 5)	*“This must be regarded as a concept for the future, as it makes continuing education alongside professional work much easier to integrate into daily life. Additionally, it allows for significantly greater flexibility in engaging with the learning content.”* (*3:20*, Supporting Information)
	(I e) Supervision 11% (*n* = 4)	*“Everything ran smoothly, and there was always a contact person available for any questions.”* (*3:13*, Supporting Information)
	(I f) Travel Distance 6% (*n* = 2)	*“This concept suited me very well, as it made it possible to complete the program despite the long distance between my place of residence and the university while simultaneously maintaining my clinical practice.”* (*3:11*, Supporting Information)
(II) Evaluated for Enhancement 14% (*n* = 5)	(II a) Ratio of Online to In‐Person Teaching 8% (*n* = 3)	*“A slightly higher proportion of in‐person sessions would have been preferable for me to allow for more practical exercises and greater interaction among participants.”* (*3:33*, Supporting Information)
	(II b) Virtual Classroom 6% (*n* = 2)	*“The virtual classroom was very good, but often very long—over 2 h in the evening after a full workday felt excessive.”* (*3:36*, Supporting Information)

The concept was widely recognised as an effective approach to work‐integrated learning. Many participants appreciated the balanced mix of online and in‐person teaching, as it allowed for both virtual and face‐to‐face interactions. Additionally, the format provided new perspectives. Another key advantage was the increased flexibility, enabling learners to better integrate their studies into their professional lives. The supervision within the program was also positively received, as there was always a contact person available. Furthermore, the concept facilitated access to education, especially for participants living far from the university. At the same time, some areas for improvement were identified. A suggestion was to increase the proportion of in‐person sessions slightly, Additionally, the duration of virtual classroom sessions was perceived as challenging, especially in the evenings after a full workday.

The majority of respondents reported positive changes in their treatment approach due to the Master program (Table 6; original results of the alumni survey are provided in S4, Supporting Information).

**TABLE 6:** Impact of the Master Program on Dental Treatment of Alumni, (*n* = absolute number of AUs, % = proportion of responses rounded to the nearest whole number, based on a total of 47 AUs).

Impact of the Master Program on Dental Treatment of Alumni		
Main category	Subcategory	Example for an AU
(I) Positively Evaluated 85% (*n* = 40)	(I a) Expansion of General Professional Competencies 23% (*n* = 11)	*“Increase in competence in handling severe and complex patient situations* (*planning, prognosis assessment, and therapy implementation*).*”* (*4:22*, Supporting Information)
	(I b) Gain in Treatment Confidence 19% (*n* = 9)	*“Nothing can throw me off track; I have gained confidence in all treatment phases.”* (*4:42*, Supporting Information)
	(I c) Improvement of Surgical Skills 15% (*n* = 7)	*“Significantly improved atraumatic surgery, resulting in fewer postoperative complaints for the patient in every surgical procedure.”* (*4:25*, Supporting Information)
	(I d) Enhancement of Periodontal Treatment 15% (*n* = 7)	*“Periodontitis treatment has become more complex, with causes being examined, identified, and treated more precisely.”* (*4:34*, Supporting Information)
	(I e) Optimization of Procedures 13% (*n* = 6)	*“More precise evaluation, improved documentation through, for example, photos, and a coordinated, structured concept.”* (*4:13*, Supporting Information)
(II) Evaluated as Challenging 15% (*n* = 7)	(II a) Challenges in the Implementation of Treatment Plans 11% (*n* = 5)	*“Sometimes one might become a bit too perfectionistic, and it is important not to forget to involve the patients and to occasionally accept a case as it turns out.”* (*4:6*, Supporting Information)
	(II b) Challenges within the Framework of Health Insurance Policy Restrictions 4% (*n* = 2)	*“The acquired competence cannot always be implemented in everyday practice due to health insurance policy restrictions.”* (*4:1*, Supporting Information)

The majority of respondents reported positive changes in their treatment approach due to the Master program. The most frequently mentioned improvement was the expansion of general professional competencies, particularly in handling complex patient cases, including planning, prognosis assessment, and therapy implementation. Additionally, many alumni noted a gain in treatment confidence, stating that they felt more secure across all treatment phases. Further benefits included an improvement in surgical skills, specifically in atraumatic surgical techniques, leading to reduced postoperative discomfort for patients. Similarly, enhancements in periodontal treatment were highlighted, with alumni reporting a more comprehensive and precise approach to diagnosis and treatment. Moreover, optimization of procedures was cited, with improvements in evaluation, documentation, and the structured organisation of treatment plans. Despite these positive developments, some respondents identified challenges in implementing their acquired skills. The most common difficulty was related to treatment planning, with some alumni noting a tendency toward excessive perfectionism, emphasising the importance of balancing high standards with practical considerations and patient involvement. Additionally, restrictions imposed by health insurance policies were mentioned as a limiting factor, making it difficult to fully apply their newly gained expertise in everyday practice.

### 3.4 Academic Achievements and Publications

**FIGURE 9:**
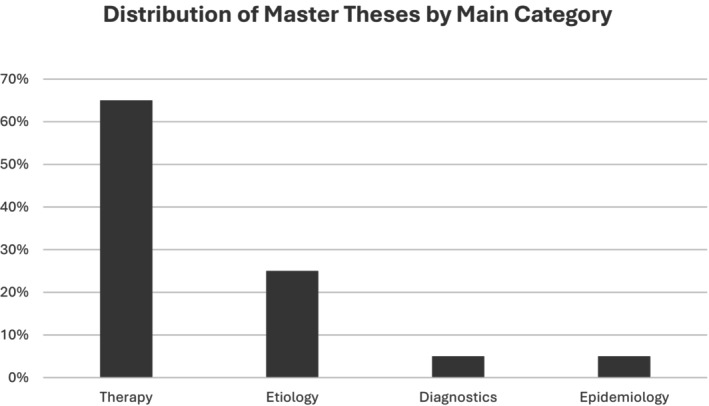
Distribution of Master Theses by main category.

A total of 96 master's theses were completed during the evaluation period. Thematic analysis revealed four main research areas: Therapy, Aetiology, Diagnostics, and Epidemiology (Figure 9).

**FIGURE 10:**
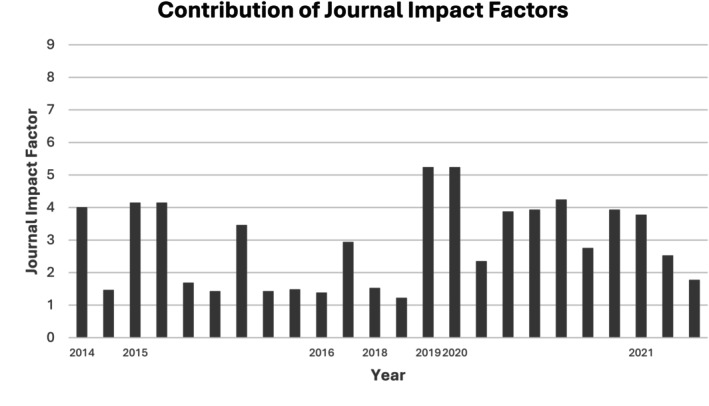
Contribution of journal impact factors by years.

Between 2014 and 2021, a total of 24 peer‐reviewed publications associated with the master's program were published (Table S5, Supporting Information). The largest group of these publications (46%, *n* = 11) addressed various aspects of periodontology, with topics ranging from implant therapy to the influence of nutrition on periodontal diseases. 29% (*n* = 7) of the publications were related to prosthodontics. This included topics such as focusing on oral rehabilitation following tooth loss or damage. Additionally, one study explored digital innovations in analgesia specifically related to pain management techniques. For the evaluation of scientific impact, the Journal Impact Factor (JIF) of the respective journal in the publication year was used. Thus, each publication was assigned the JIF valid for its journal in the year of appearance. The cumulative JIF of all publications was 70.03. The median JIF was 2.85, with values ranging from 1.23 to 5.24 (Figure 10; List of all publications and Journal Impact factors can be found in Table S6, Supporting Information).

Notably, a publication on the practice‐based research network, developed through the Master's Program, highlighted the development of a digital method to retrieve, extract, and analyse clinical data from dental practices, involving 6301 patients across nine different practices [17]. This study underscored the ability of practice‐based networks to generate substantial clinical data directly from the field of dental care and demonstrated how the Master's Program can contribute to advancing dental practice. In terms of academic career progression, two alumni have completed habilitation or hold an adjunct professorship, two are currently pursuing habilitation, and three have been employed as university teaching staff—either full‐time or part‐time—at various points in their careers.

### 3.5 Awards and Recognition

The Master's Program in Periodontology and Implant Therapy, along with its faculty members, has received multiple awards recognising its innovation and contributions to dental education.

In 2010, the Thieme Innovation Award was granted for the program's outstanding didactic concept. This was followed in 2012 by the Instructional Development Award (IDA) for the Parocase project, which integrated real clinical cases into self‐directed learning. The project aimed to enhance diagnostic reasoning and therapeutic decision‐making by linking theory to clinical reality. In 2013, both the program director and the deputy program director were awarded the Excellence in Dental Education Award by the Association for Dental Education in Europe (ADEE), recognizing their achievements in postgraduate curriculum development and teaching innovation. Additionally, the deputy program director received separate recognition for the integration of motivational interviewing into the curriculum. This educational innovation improved communication training and supported more effective patient behavior change, particularly in the context of oral hygiene instruction.

## 4 Discussion

The present study aimed to identify factors contributing to the successful establishment of the postgraduate master's program in Periodontology and Implant Therapy at the University of Freiburg over the past 15 years through a multiperspective analysis. Structural adjustments made over the years, including continuous content updates and user‐oriented modifications, such as regular content updates, optimization for smartphone compatibility, and improved accessibility across devices, seemed to play a crucial role in the program's endurance. This finding supports the notion that continuous improvement and adaptability are essential components of effective educational programs [18]. Furthermore, the program's consistent reaccreditation by ACQUIN (ACQUIN e.V., Germany) since its initial approval in 2007 reflects its alignment with evolving professional standards, reinforcing its academic standing and credibility.

### 4.1 Participant's Socidemopgraphic Characteristics

At programme entry, participants represented a broad range of age groups. This age diversity indicates that the master's program was able to attract participants from a broad range of age groups, reflecting its accessibility and appeal to dentists at different career stages. This diversity in age distribution is particularly noteworthy given the predominantly digital, blended‐learning format of the curriculum. The sociodemographic data showed that the program successfully reached participants from diverse geographical backgrounds. Out of a total of 106 graduates, 16 came from abroad, representing approximately 15% of the cohort. Although primarily nationally oriented, the blended‐learning design seems to have attracted international participants. The international students primarily originated from geographically close Switzerland, followed by Austria, the Netherlands, and Norway. This reach may be attributed to the advantage of digital asynchronous formats, which offer location‐independent individual learning opportunities [19]. It's blended‐learning design appears to have also appealed to international students. By ensuring platform independence and reducing access barriers, the reach of educational programs can be significantly expanded beyond the boundaries of one's own faculty [20]. Findings from this study suggest that blended learning enhances the accessibility of dental education, particularly for students from rural areas, individuals with family responsibilities, and full‐time or self‐employed professionals. The reduced need for in‐person attendance was frequently cited as a crucial factor in making postgraduate education more feasible, as it minimised travel demands and enabled participants to balance their studies with professional and personal obligations. This flexibility appears to be a key advantage of digital asynchronous learning, which allows for location‐independent, individualised study opportunities. This finding is consistent with previous studies that highlight the advantages of blended learning in accommodating the busy schedules of healthcare professionals [21, 22]. The overrepresentation of participants from rural areas further underscores the potential of digital education in bridging gaps in access to CPD, particularly in less urbanised regions. These findings align with previous reports which emphasises that blended learning models provide flexible educational opportunities, particularly for students in remote regions [23]. In contrast, participants from medium‐sized towns were underrepresented, while those from small and large cities corresponded to the average number of realised dental practice locations. While these insights provide valuable correlations, it should be noted that causal relationships cannot be established based solely on demographic data.

### 4.2 User Perspective on Learning Experience and Digital Format

Survey responses indicated a predominantly positive reception of the blended‐learning format. The virtual classroom was recognised for fostering continuous engagement, structured learning, and time discipline, whereas face‐to‐face sessions were perceived as highly productive and essential for hands‐on training. Despite the benefits of digital learning, some participants expressed a need for more practical, hands‐on training, highlighting the challenge of fully translating theoretical knowledge into clinical practice in digitally led programs. The demand for self‐organisation in asynchronous learning formats was perceived as both empowering and demanding, aligning with studies that suggest that online format's demands for a high level of self‐organisation [24]. Moreover, virtual evening sessions were sometimes experienced as stressful, emphasising the need to balance digital education with personal time management. This dual nature of flexibility—both as a facilitator and a source of strain—mirrors broader tensions faced by professionals balancing work, education, and family life. However, it does not resolve the issue of limited time resources for working students but merely shifts it. Flexibility in the learning process can thus be seen as both beneficial and challenging, especially for full‐time employees with family responsibilities. Some students report difficulties in managing their academic workload despite it being within a typical study framework, reflecting a broader trend in the medical profession, where conflicts between work, studies, and family persist despite existing family‐oriented measures [25]. For working dentists, limited time creates tensions between professional development, work commitments, and private life. While blended learning enhances accessibility to postgraduate education, survey results indicate that self‐directed time management does not fully mitigate this tension. Despite technological acceleration saving time, it paradoxically contributes to an overall scarcity of time resources, underscoring that acceleration alone is insufficient to explain changes in contemporary time perception [26].

### 4.3 Didactive Perspective, Faculty Support and Technological Integration

Participants also valued the strong faculty support and favorable student‐to‐instructor ratio, which contributed to a supportive and effective learning environment. Indeed, existing literature suggests that personalised support and mentorship are critical for the success of professional education programs [27, 28]. The program's success in integrating digital platforms demonstrated a significant achievement in contemporary education systems, where technological readiness can influence educational outcomes. The blended learning approach corresponds to current developments in education and thus reflects demands from the wider societal context [29]. The shift to online learning platforms during the COVID‐19 pandemic highlighted the program's resilience and adaptability, traits that are essential for modern educational institutions facing similar unexpected challenges. The flexibility offered by such models can provide substantial benefits in terms of accessibility and inclusivity. The positive experiences reflect findings from other disciplines where it was shown that e‐learning is often considered as flexible [30] and working online from home positively affected work‐life balance [31].

### 4.4 Scientific Output and Clinical Orientation

The academic output associated with the program further underscores its institutional value. A total of 96 master's theses—predominantly clinically oriented—and 24 peer‐reviewed publications emerged during the study period. These findings indicate that the program not only promoted clinical competence but also facilitated scholarly activity. This aligns with studies highlighting the potential for professional training programs to contribute to academic research and innovation [32]. The strong clinical focus of the master's thesis may be attributed not only to the fact that all participants were already practicing clinicians but also to the structure of the program itself, which appears to prioritize clinical work. The program's blended‐learning design enabled participants to pursue postgraduate education without interrupting their professional activities, thereby fostering research topics that are both clinically relevant and grounded in real‐world treatment settings. By integrating academic learning with participants' ongoing professional practice, the program has actively facilitated clinically oriented research topics for master's theses, reinforcing its application‐driven approach to postgraduate education. Given the increasing emphasis on multidisciplinary collaboration in both clinical practice and academia, the potential influence of blended learning formats on collaborative learning processes warrants consideration. While digital formats offer logistical advantages, they may reduce spontaneous professional interactions that typically arise in face‐to‐face settings. However, in the context of periodontology, blended learning also enables a valuable integration of academic education and clinical routine. Unlike studies conducted in controlled clinical environments, practice‐integrated research enables context‐specific insights generated under routine care conditions, enhancing external validity and clinical applicability. Blended learning formats, by facilitating the parallel engagement in academic study and clinical practice, offer a unique framework for generating evidence that reflects the complexity of everyday treatment settings.

### 4.5 Implications for Future Program Design

Although periodontology, as a clinically intensive and highly manual discipline, has traditionally relied on face‐to‐face teaching, the present findings demonstrate that blended learning can serve as a viable and effective postgraduate training model.

The program's modular, digitally supported structure, integration of mentoring, and targeted in‐person sessions proved compatible with the realities of clinical work, enabling participants to combine advanced training with patient care. Embedding academic projects into routine clinical activity further promoted context‐sensitive, practice‐relevant research.

Based on these results, future programs can adopt similar structural elements and implement regular participant surveys to continuously assess needs, satisfaction, and perceived learning outcomes. Such feedback‐driven evaluation enables iterative curricular adaptation, ensures alignment with the evolving requirements of clinical practice, and supports evidence‐based optimization of postgraduate dental education.

### 4.6 Limitations

Several limitations of this study must be acknowledged. First, the qualitative content analysis used—while appropriate for exploratory studies—was conducted by a single coder, which introduces potential subjectivity. Enhancing objectivity and reproducibility could have been achieved by employing second and third coders to cross‐verify the results [15]. Due to the extensive nature of the data, a more elaborate coding process was avoided to maintain clarity. Second, the user survey method, while cost‐effective and capable of reaching respondents with busy schedules, may limit participants' ability to ask clarifying questions during the survey process [33]. Third, the survey's voluntary nature may have led to selection bias, as participants with particularly strong positive or negative experiences may have been more likely to respond. Fourth, only about 30% of alumni participated, and the representativeness of the responses for the full graduate cohort remains uncertain. Last but not least, the findings reflect the experiences and institutional framework of a single master's program within the German higher education system. Therefore, the extent to which these results can be generalised to continuing professional development (CPD) programs in other geographical, cultural, institutional, or regulatory contexts remains uncertain.

## 5 Conclusion

This multiperspective analysis of the Periodontology Master's program at the University of Freiburg identified the blended‐learning format and faculty support as key determinants of its long‐term success. The program's structure facilitated access to postgraduate education for diverse student groups, including those from rural areas, individuals with family responsibilities, full‐time professionals, and self‐employed practitioners. The efficiency and flexibility of the blended‐learning approach allowed participants to balance professional, academic, and personal commitments while minimising travel obligations. The combination of online and in‐person learning was widely regarded as beneficial, with the virtual classroom supporting structured learning and continuous engagement, while face‐to‐face sessions were highlighted for their productivity and hands‐on training opportunities. The program was credited with enhancing clinical competencies, particularly in complex treatment planning, surgical skills, and periodontal therapy, leading to improved patient outcomes. Alumni reported increased treatment confidence and a more structured approach to evaluation, documentation, and workflow optimization. Furthermore, the analysis showed that the master's program generated its own scientific output and led to scientific innovations such as a practice‐based research network. In summary, it was shown that the combination of online study, coordinated personal support, and on site phases significantly contributed to the long‐term successful establishment of the blended learning master's program.

## Funding

The authors have nothing to report.

## Conflicts of Interest

The authors declare no conflicts of interest.

## Supporting information


**Table S1:** Survey Results from March 2021—Graduates' Positive Evaluations of the Postgraduate Master's Program in Periodontology and Implant Therapy at the University of Freiburg, Categorised into Main and Subcategories.
**TABLE S2:** Survey Results from March 2021—Graduates' Negative Evaluations of the Postgraduate Master's Program in Periodontology and Implant Therapy at the University of Freiburg, Categorised into Main and Subcategories.
**TABLE S3:** Survey Results from March 2021—Graduates' Experiences with the Blended‐Learning Format in the Master's Program in Periodontology and Implant Therapy at the University of Freiburg.

## Data Availability

The data that support the findings of this study are available from the corresponding author upon reasonable request.
