# Supplementary material for: A 15‐Year Case Study of the Structures and Outcomes in a Blended Learning Postgraduate Master Programme for Periodontology and Implant Therapy
Source: Eur J Dent Educ. 2026 Feb 21;30(3):1352–68. doi: 10.1111/eje.70091 (PMC13383306; doi:10.1111/eje.70091)
Supplement: Supplementary file 1 — Table S1: Survey Results from March 2021—Graduates' Positive Evaluations of the Postgraduate Master's Program in Periodontology and Implant Therapy at the University of Freiburg, Categorised into Main and Subcategories. TABLE S2: Survey Results from March 2021—Graduates' Negative Evaluations of the Postgraduate Master's Program in Periodontology and Implant Therapy at the University of Freiburg, Categorised into Main and Subcategories. TABLE S3: Survey Results from March 2021—Graduates' Experiences with the Blended‐Learning Format in the Master's Program in Periodontology and Implant Therapy at the University of Freiburg. [file EJE-30-1352-s001.docx]

## **Supplementary**

## **Original Results of the Alumni-Survey**

## **1 |** Was beurteilen Sie an der postgradualen Fortbildung im Master Parodontologie und Implantattherapie der Universität Freiburg als positiv?

| **Kategorie** | **Subkategorie** | **Aussage** |
| --- | --- | --- |
| Lehrpersonal  N=21 | Betreuungsver-hältnis  N=7 | 1. gutes Betreuungsverhältnis |
|  |  | 1. Kleine Gruppengröße |
|  |  | 1. Sehr angenehme Atmosphäre, nicht zu große Gruppen |
|  |  | 1. Kleine Gruppen |
|  |  | 1. Positiv war auch die kleine Gruppe der Teilnehmer/innen |
|  |  | 1. Intensive Betreuung |
|  |  | 1. gute Betreuungssituation, familiäre Lernatmosphäre |
|  | Referent*innen  N=6 | 1. tolle Referent*innen |
|  |  | 1. tolle Referenten; tolle Betreuung |
|  |  | 1. Sehr renommierte Dozenten; Hohe Wissensvermittlung |
|  |  | 1. Strukturiert, sehr gute Referenten aus allen Fachbereiche |
|  |  | 1. Verschiedene Sichtweisen auf Behandlungen, weil viele   Dozenten aus Praxen |
|  |  | 1. viele verschiedene Referenten aus verschiedenen Unis., alle   Themen der Parodontologie wurden bearbeitet |
|  | Fachbereichs-leitung  N=3 | 1. angenehme und wertschätzende Betreuung durch Frau Prof. Ratka-   Krüger |
|  |  | 1. Fr Prof Ratka kümmert sich persönlich, sehr gute Vortragende |
|  |  | 1. tolle leitende Professorin |
|  | Teletutoren  N=2 | 1. fachliche Ansprechpartner: Teletutoren |
|  |  | 1. gute Erreichbarkeit und Kompetenz der Teletutoren |
|  | Team  N=3 | 1. Lernen von angesehenen Fachleuten |
|  |  | 1. kompetentes Team in Freiburg |
|  |  | 1. Hochqualifizierte Lehrkräfte |
| Inhalt  N=21 | Allgemeine Fachinhalte  N=13 | 1. umfangreiches Lehrmaterial   interessante fachliche Aspekte und "Nebenschauplätze" durch die Module Notfall und Kommunikation |
|  |  | 1. Zugang zur wissenschaftlichen Literatur |
|  |  | 1. Zugriff auf Literatur und Möglichkeiten eigener Fallpräsentationen |
|  |  | 1. Bandbreite der Themen |
|  |  | 1. Quantität der Lehrmaterialien |
|  |  | 1. Aneignung Fachwissen |
|  |  | 1. Zugriff auf wiss. Datenbanken |
|  |  | 1. besseres Verständnis von Paro, objektivere Sicht auf ZM allgemein, besseres Verständnis der Biologie |
|  |  | 1. aktuelle Lehrinhalte |
|  |  | 1. man ist immer auf dem aktuellsten Stand |
|  |  | 1. Erweitert den Horizont |
|  |  | 1. fachliche Bewusstseinserweiterung; Abdeckung zahlreicher nicht-paro-Themen, Update in ZHK |
|  |  | 1. sehr gute wissenschaftl. Potenz der Uni Freiburg |
|  | Anwendbarkeit  N=4 | 1. praxisnahe Wissensvermittlung |
|  |  | 1. Das parodontale Gesamtkonzept war überzeugend und ist immer noch vollständig im Praxisalltag integriert |
|  |  | 1. Die Vermittlung eines fachlich hochwertigen und kohärenten PARO-Behandlungskonzeptes mit hohem Praxisbezug |
|  |  | 1. Umsetzbare Konzepte für den Praxisalltag |
|  | Chirurgie  N=4 | 1. Chirurgie enthalten |
|  |  | 1. Erlernen Operationstechniken |
|  |  | 1. praktische Woche in der wir uns komplett auf die Chirurgie konzentrieren konnten, toll war es auch unsere operierten Patienten nach einem halben Jahr wieder zu sehen |
|  |  | 1. Arbeit an Humanpräparaten |
|  |  |  |
| Konzept  N=36 | Blended Learning  N=21 | 1. Kombination Theorie und Praxis |
|  |  | 1. Kombinierbarkeit mit Berufsalltag, Praxisnähe |
|  |  | 1. online-Konzept |
|  |  | 1. Gute Lernplattform Umfangreiche Wissenserweiterung ohne allzu viele Präsenztermine |
|  |  | 1. freie Zeiteinteilung durch Online-Studium |
|  |  | 1. Die für den postgradual Studierenden zeitschonende Verbindung von Online- und Präsenzlehre |
|  |  | 1. Kombination online/Präsenz, Inhalte umfassend behandelt |
|  |  | 1. frühzeitig ein gutes Konzept der online Präsentation und Weiterbildung |
|  |  | 1. Berufsbegleitend möglich, am aktuellen Stand, sehr gut organisiert,, kleine Gruppe in der man viel lernt, |
|  |  | 1. Sehr gute Organisation, normaler Arbeitsalltag möglich, geringerer Aufwand Reise nur bei Präsenzveranstaltung notwendig |
|  |  | 1. sehr gute Vereinbarkeit von Beruf und Studium, sehr gute Vereinbarkeit mit Familie |
|  |  | 1. Aufteilung des Lehrstoffes in Online und Präsenz, ausgezeichnete Dozenten, regelmäßige Online-Meetings in gleichbleibenden Studiengruppen |
|  |  | 1. Onlineangebot für die theoretischen Inhalte. Da diese alle zwei Wochen stattfinden könnten, hatte man kürzere Intervalle zum vertiefen der Themen. Das gewährleistet einen langfristigen Lernerfolg |
|  |  | 1. viele Inhalte wurden online vermittelt, dadurch geringer Praxisausfall und flexible Zeiteinteilung möglich |
|  |  | 1. Hoher Online-Anteil; Wenig Praxisausfall; |
|  |  | 1. Bequemes Lernen von Zuhause/Online |
|  |  | 1. gewohntes Format |
|  |  | 1. Lernplattform |
|  |  | 1. Nutzung elektron. Medien |
|  |  | 1. Die Online-Gestaltung erlaubt ein zeitlich selbstbestimmtes Studieren |
|  |  | 1. Online Plattform, dadurch Integration in Beruf und Familie möglich |
|  | Präsenzphasen  N=6 | 1. Die Fortbildung ist sehr umfangreich und vielseitig. Insbesondere die Präsenzzeiten sind in guter Erinnerung und äußerst fruchtbar gewesen |
|  |  | 1. Gute Strukturierung praktischer Teil |
|  |  | 1. Die Präsenzphasen für die praktischen Übungen u.a. waren jedes Mal fachlich und menschlich ein Highlight!. |
|  |  | 1. interessante praktische Teile, gute Verpflegung, nettes Abendprogramm, Gefühl immer dran zu sein mit den 2-wöchigen Klassenzimmern |
|  |  | 1. Intensive Präsenzveranstaltungen in Freiburg |
|  |  | 1. intensive Präsenzveranstaltungen, z. B. in der Chirurgie |
|  | Virtuelles Klassenzimmer  N=4 | 1. regelmäßige Fallbesprechungen, Vorlesungen Regelmäßigkeit |
|  |  | 1. regelmäßige Motivation durch virtuelle Klassenzimmer, guter Austausch dadurch möglich |
|  |  | 1. Das Tool und die Inhalte des Virtual Classrooms (VC) und der dadurch mögliche Wissensaustausch der Studierenden auch untereinander |
|  |  | 1. Begleitung vom virtuellen Klassenzimmer mit Aufabenstellungen, was die zeitliche Disziplin sicher stellt und den menschlichen Kontakt mit Mitstudierenden und Dozenten lebendig hält. |
|  | Aufbau  N=3 | 1. fundierte Weiterbildung mit "rotem" Faden |
|  |  | 1. Strukturierter Aufbau |
|  |  | 1. Organisation |
|  | Postgradual  N=2 | 1. Koordinierter postgraduierten Lehrgang |
|  |  | 1. Kontinuum |
| Vernetzung  N=7 | Austausch Studierende  N=7 | 1. Austausch Fachwissen mit Kollegen |
|  |  | 1. kollegialer Austausch in kleinen Gruppen |
|  |  | 1. Nette Studienkollegen in ‚Feuerzangenbowlenatmosphäre‘ |
|  |  | 1. Netzwerk-Aufbau mit Kollegen |
|  |  | 1. Kontakt mit fachlich überdurchschnittlich interessierten Kollegen |
|  |  | 1. Austausch unter den Studierenden |
|  |  | 1. kollegiales Miteinander in den Präsenzphasen |

Tab. S1: Survey Results from March 2021 – Graduates’ Positive Evaluations of the Postgraduate Master’s Program in Periodontology and Implant Therapy at the University of Freiburg, Categorized into Main and Subcategories

## **2 |** Was beurteilen Sie an der postgradualen Fortbildung im Master Parodontologie und Implantattherapie der Universität Freiburg als negativ?

| **Kategorie** | **Subkategorie** | **Aussage** |
| --- | --- | --- |
| Inhalt  N=19 | Anwendbarkeit  N=3 | 1. Stellenweise sehr wissenschaftliche Vorträge ohne Nutzen für die Praxis. |
|  |  | 1. Manches Verfahren erschien im Nachhinein ein bisschen zu exotisch und wirklichkeitsfremd |
|  |  | 1. Anfangs allgemein Theorie- und Basics-lastig. Zu wenig praktische Paro-Basics für Paro-Wiedereinsteiger bzw. -Anfänger. |
|  | Chirurgie  N=5 | 1. Chirurgie am Patienten (Supervision): einerseits ein sehr gelungenes Modul, andererseits wäre natürlich immer noch mehr wünschenswert, z. B. eine weitere OP. Es ist aber klar, dass dadurch der Mehraufwand sehr   stark wachsen würde |
|  |  | 1. Chirurgische Therapie war unterrepräsentiert; ich hätte gern mehr den Spezialisten bei OP's zugesehen (reg. Chir. oder plastische PA Chirurgie) |
|  |  | 1. Die Möglichkeiten, operative Eingriffe erlernen zu können, sind naturgemäß limitiert und könnten z.B. durch OP-Videos und/oder Hospitationen ergänzt werden. |
|  |  |  |
|  |  | 1. Ggf. waren die unterschiedlichen Chirurgietechniken nicht ganz ausgewogen im Programm repräsentiert (z. B. resektiv —> kam vielleicht etwas zu kurz) |
|  |  | 1. das Modul Implantologie mit seiner Präsenz war zu oberflächlich |
|  | Allgemeine Fachinhalte  N=7 | 1. überarbeitungsbedürftige Lernmaterialien |
|  |  | 1. Es wurden zahlreiche Nicht Parodontologie assoziierte Themen behandelt. Kein Kuerettenschleifkurs damals |
|  |  | 1. Zu intensiver Grundlagenteil |
|  |  | 1. Teilbereich nichtchirurgische Therapie kam in praktischer Hinsicht etwas   zu kurz |
|  |  | 1. Wenig Update |
|  |  | 1. Aktualität der Module war nicht immer gegeben, Fehler wurden nicht in den Skripten geändert |
|  |  | 1. Schade (aber einsehbar) ist es, dass nach Abschluss des   Studienganges kein Zugriff mehr auf Literaturdatenbanken möglich ist.Auftretende Kritikpunkte wurden im Verlauf des Studienganges mit Dozenten und Organisatoren ausführlich thematisiert und diskutiert und  auch weitgehend angepasst. An die einzelnen Inhalte dieser Punkte kann ich mich allerdings nicht mehr erinnern, sorry. |
|  | Abrechnung  N=2 | 1. Die Information über die verschiedenen Leistungen in Bezug auf Abrechenbarkeit und Erstattungsmöglichkeiten waren eher dürftig. |
|  |  | 1. Behandlung wirtschaftlicher Aspekte in der PA fehlte mir |
|  | Periimplantitistherapie  N=2 | 1. Periimplantitistherapie fehlte mir |
|  |  | 1. wenig Infos zu Paro an Implantaten |
| Konzept  N=19 | Aufbau  N=3 | 1. Wir Frühbucher wurden leider nicht mehr in manche Konzepte mit integriert. |
|  |  | 1. Relation Zeitaufwand und Neuwissen |
|  |  | 1. Da wir der erste Studiengang waren und viele der Teilnehmer extrem gut qualifiziert, konnte man deutlich bemerken, dass einige Veränderung in der Organisation nötig waren. Das war jedochnicht nur negativ, da durch Diskussion und kollegiale Gespräche versucht wurde, für alles eine Lösung zu finden. |
|  | Anforderungen  N=6 | 1. Wissenschaftlich hoher Anspruch Zeitaufwand Masterarbeit und Patientenfälle |
|  |  | 1. viele Hausübungen/schwierig mit Vollzeitbeschäftigung-Kinder, entspricht aber natürlich dem Umfang eines Studiums (im direkten Vergleich mit anderen Mastern allerdings eindeutig viel mehr Aufwand) |
|  |  | 1. Es war am Anfang sehr schwierig zu verstehen wie man eine   Masterthese schreibt wenn man so etwas noch nie gemacht hat. |
|  |  | 1. Multiple choice tests |
|  |  | 1. Fotografieren ist nicht so meins |
|  |  | 1. die Kosten limitierend und schon eine große Investition, der Zeitaufwand ist beträchtlich |
|  | Präsenzphasen  N=9 | 1. teilweise unnötige Fortbildungen in Präsenz |
|  |  | 1. Etwas zu wenig Praxis |
|  |  | 1. Eigentlich nur der lange Anfahrtsweg nach Freiburg; dies ist natürlich ein rein subjektiver Faktor |
|  |  | 1. Für mich die relativ weite Anreise. |
|  |  | 1. Zu wenig Praxis |
|  |  | 1. Wenig praktische Übungen |
|  |  | 1. wenig praktische Erfahrungen |
|  |  | 1. Begrenzte Möglichkeit der praktischen Weiterbildung |
|  |  | 1. Zu wenig praktische Übung an Modell, Schwein, Patient. |
|  | Virtuelles Klassenzimmer  N=1 | 1. Die VCS waren am Anfang nicht so effektiv, dass sie mir geholfen   hätten. |
| Verneinung der Frage  N=5 | Nichts  N=4 | 1. Nichts |
|  |  | 1. eigentlich ist nichts negativ, wenn man so will. |
|  |  | 1. Ich kann nichts als negativ beurteilen. |
|  |  | 1. bisher nichts und das ist eine ernstgemeinte Aussage |
|  | Wenig negatives  N=1 | 1. insgesamt wenig Negatives |
| Netzwerk  N=2 | Austausch  Studierende  N=2 | 1. Gemeckere und Gezicke von Mitstudierenden, wenn es um das   Thema Mittagessen oder Pünktlichkeit und dgl. geht |
|  |  | 1. kein regelmäßiger Austausch |
| Lehrpersonal  N=2 | Betreuungsverhältnis  N=1 | 1. Betreuung war nicht durchgehend gut, Fall-Korrekturen haben sich in die Länge gezogen |
|  | Referent*innen  N=1 | 1. einzig , dass ein , zwei Referenten nicht aktuell mit Ihren Vorträgen waren, bzw. in der Auswahl der Prüfungsfragen total daneben lagen |

Tab. S2: Survey Results from March 2021 – Graduates’ Negative Evaluations of the Postgraduate Master’s Program in Periodontology and Implant Therapy at the University of Freiburg, Categorized into Main and Subcategories

**3 |** Wie waren Ihre Erfahrungen mit dem Blended-Learning-Format?

| **Kategorie** | **Subkategorie** | **Aussage** |
| --- | --- | --- |
| Positiv  N=31 | Allgemeines Lob  N=9 | 1. Eine große Stärke des Studiengangs. |
|  |  | 1. Gut |
|  |  | 1. Die Erfahrungen waren durchweg positiv. |
|  |  | 1. Hervorragend |
|  |  | 1. Sehr gut |
|  |  | 1. Super |
|  |  | 1. Insgesamt gut |
|  |  | 1. Sehr gut, im Angesicht der "Corona-Problematik" besonders wertvoll |
|  |  | 1. sehr guter Ansatz für berufsbegleitendes Lernen |
|  | Anreise  N=2 | 1. Zeitersparnis durch Wegfall der Wege/Anreise |
|  |  | 1. Mir kam dieses Konzept sehr entgegen, da dadurch die Realisierung des Studienganges trotz der weiten Entfernung zwischen Wohnort und Universität bei parallel möglicher Aufrechterhaltung der Praxistätigkeit erst möglich wurde. |
|  | Betreuung  N=4 | 1. Sehr gut dank top Support. |
|  |  | 1. alles lief reibungslos, bei Fragen gab es immer einen Ansprechpartner |
|  |  | 1. Sehr positiv, reibungsloser Ablauf wegen guter Betreuung |
|  |  | 1. Dank der ständigen Mitarbeit von Milena und Stefan waren etwaige Hänger auch immer schnell behoben |
|  | Zeitliche Souveränität  N=5 | 1. Prima, das macht zeitlich unabhängig und man kann lernen wann man will. |
|  |  | 1. Sehr gute Möglichkeit der Einteilung. Abendunterricht kollidiert nicht mit Praxisalltag. Gut mit Familie vereinbar. Man kann sich die Inhalte auswählen |
|  |  | 1. durch die Onlineangebote war man sehr flexibel |
|  |  | 1. Wenig Praxisfehltage |
|  |  | 1. Dies muss als Konzept für die Zukunft gesehen werden; so ist eine berufsbegleitende Weiterbildung sehr viel einfacher in den Alltag integrierbar; ausserdem kann man sich zeitlich sehr viel flexibler mit den Lerninhalten auseinandersetzen |
|  | Verhältnis Online- / Präsenzlehre  N=6 | 1. Sehr gut. Gute Vorbereitung auf die Präsenzveranstaltung mittels Online Angebote und Vorlesung; Gut ausgearbeitete Unterlagen |
|  |  | 1. Präsenzveranstaltungen sehr wichtig für praktische Übungen und Erfahrungsaustausch. Onlinevorlesungen vor allem für trockene Theorie sehr praktisch weil nicht zu viel am Stück |
|  |  | 1. Super, nur so war es überhaupt möglich die Weiterbildung auf diesem Niveau durchzuführen |
|  |  | 1. Es war die erste Erfahrung und diese war sehr positiv, die interaktiven Inhalte waren verständlicherweise am besten |
|  |  | 1. Man erhält gleichzeitig Kontakt online und in der Präsenz um Fragen zu stellen oder Stoff zu vertiefen. Das Verhältnis war sehr ausgewogen. |
|  |  | 1. Sehr gut, Selbststudium hat gut funktioniert, Präsenz ist gut getaktet |
|  | Perspektiv Zugewinn  N=5 | 1. davor nie Kontakt damit, Platform Adobe neu; so begeistert, dass das Konzept Adobe von der ZÄK Vorarlberg für den Unterricht und die Fortbildung übernommen wurde! Besonders in Zeiten von Corona hat sich das bestätigt und bewährt |
|  |  | 1. ich bin absolut kein Computer Spezialist. Aber selbst ich habe viel gelernt und kam gut zurecht |
|  |  | 1. Sehr gut! Anders wäre es neben dem Abruf gar nicht möglich und ich habe auf diese Weise auch eine andere PC Kompetenz erworben als ich davor hatte! |
|  |  | 1. Ich habe dies zum ersten Mal in dieser Form erlebt und finde es eine sehr ansprechende, moderne und effiziente Art der Lehre. |
|  |  | 1. hätte nie gedacht, dass mir das in meinem fortgeschrittenen Alter noch so viel Spaß macht. In 98% der Fälle gab es eine gute Verfügbarkeit und Konstanz bei den Online-Modulen |
| Verbesserungswürdig  N=5 | Verhältnis Online- / Präsenzlehre  N=3 | 1. Ggf. hätten einige der Präsenzveranstaltungen noch effektiver genutzt werden können für Hands-on- Formate, dafür hätte ein Teil der Theorie ggf. doch in das VC verlagert werden können |
|  |  | 1. Etwas mehr Präsenz hätte mir besser gelegen, um mehr praktische Übungen und mehr Austausch untereinander zu schaffen. |
|  |  | 1. habe lieber nur Präsenz oder nur online |
|  | Virtuelles Klassenzimmer  N=2 | 1. Im VC kein guter Austausch möglich, oft gar keine Meldungen seitens des Publikums |
|  |  | 1. Virtuelles Klassenzimmer war auch sehr gut aber oft sehr lange, über 2 Stunden am Abend nach einem langen Arbeitstag war dann zu lang |

Tab. S3: Survey Results from March 2021 – Graduates’ Experiences with the Blended-Learning Format in the Master’s Program in Periodontology and Implant Therapy at the University of Freiburg

## **4 |** Was hat sich Ihrer Erfahrung nach durch die Teilnahme am Master in ihrer Behandlung zum Positiven oder Negativen geändert?

| **Kategorie** | **Subkategorie** | **Aussage** |
| --- | --- | --- |
| Negativ  N=7 | Herausforderungen im Rahmen kassenpolitischer Restriktionen  N=2 | 1. Die erworbene Kompetenz ist im Praxisalltag aufgrund kassenpolitischer Restriktionen nicht immer umsetzbar |
|  |  | 1. Es wurde immer klarer, daß die PA in den Gebührenordnungen massiv zu gering bewertet ist und daher auch in Zukunft eine Nischendasein fristen wird. Dagegen wird nicht wirklich etwas unternommen und auch der Studiengang bietet keine Hilfe |
|  | Herausforderungen bei der Umsetzung von Behandlungsplänen  N=5 | 1. Anfangs sehr zeitintensiv |
|  |  | 1. Patienten-Compliance dadurch erschwert/ Behandlung in diesem Umfang oft nicht gewünscht |
|  |  | 1. viel Freizeit geopfert |
|  |  | 1. Manchmal ist man vielleicht schon etwas zu perfektionistisch und man darf nicht vergessen, die Patienten mitzunehmen und muss auch mal einen Fall so stehen lassen, wie er herauskommt |
|  |  | 1. Mitarbeiterinnen konnte ich zum Teil fachlich nicht mitnehmen, verharren in alten Mustern- ein weiter Weg liegt vor uns! |
| Positiv  N=40 | Optimierung von Behandlungsabläufen  N=6 | 1. Behandlungskonzept wird immer ausgefeilter, damit besser planbar |
|  |  | 1. Ich bin nun mit einem richtigen Konzept gerüstet und |
|  |  | 1. allgemein klarere Struktur in der Behandlungsplanung |
|  |  | 1. Koordiniertes arbeiten |
|  |  | 1. Nur positiv... Kleinigkeiten in den Behandlungsalltag mitgenommen, ohne das gesamte eigene Konzept in Frage zu stellen. Bereicherung der bisher gefahrenen Behandlungsstrategie |
|  |  | 1. Genauere Evaluation, bessere Dokumentation durch z.B. Fotos, abgestimmtes auf sich aufbauendes Konzept |
|  | Erweiterung allgemeiner fachlicher Kompetenzen  N=11 | 1. Fachlicher Hintergrund viel ausgeprägter |
|  |  | 1. Fachwissen wieder umfänglich aktualisiert, Inspirationen durch Erfahrungsaustausch mit anderen Kollegen/innen, Bereicherung der einschlägigen Fertigkeiten durch neue oder sehr spezialisierte Techniken.; Negative Änderungen kann ich nicht erkennen. |
|  |  | 1. Meine Behandlung basiert schon immer auf dem Freiburger Konzept. Meine Behandlung hat sich aber bereichert um viele Methoden. Wissen, neueste Techniken. neue Medikationen und anderes |
|  |  | 1. Zahlreiche Verbesserungen in allen zahnärztlichen Teilgebieten |
|  |  | 1. Ein paar neue Sachen gelernt |
|  |  | 1. Erweiterung des eigenen Behandlungsspektrums |
|  |  | 1. souveräneres Umgehen im Bereich der Online-Fortbildungen und im Einsatz der entsprechenden Medien |
|  |  | 1. Wunderschönes Diplom für das Wartezimmer |
|  |  | 1. Kompetenzsteigerung im Umgang mit schweren und komplexen Patientensituationen (Planung, Prognosestellungen, Durchführung der Therapie) |
|  |  | 1. durchweg Positives |
|  |  | 1. bessere und klarere Kommunikation mit den Patienten |
|  | Verbesserung der chirurgischen Fähigkeiten  N=7 | 1. Deutlich verbesserte atraumatische Chirurgie; Daher weniger postoperative Beschwerden für den Patienten bei jedem operativen Eingriff |
|  |  | 1. Die Art der Präparation; ; Biol. Breite wird eingehalten; ; mehr PA-Chir und chir. Kronenverl.; ; PA Konzept kommt Pat. zu gute |
|  |  | 1. Ich arbeite in einer Überweisungspraxis Oralchirurgie. Ich habe meinen chirurgischen Standpunkt schärfen können und bin kompetenter Ansprechpartner für die ZÄ in Sachen Paro. Das war sehr positiv. |
|  |  | 1. Operatives Vorgehen wird besser umgesetzt |
|  |  | 1. Qualität der OP-Techniken |
|  |  | 1. vor allem aber bin ich in der Chirurgie mutiger geworden und sicherer in der Beurteilung ihrer Indikation. |
|  |  | 1. Bessere Kenntnisse va PA Chirurgie, Grenzen der PA |
|  | Verbesserung der Parodontalbehandlung  N=7 | 1. Bin bei Pa konsequenter geworden ; Mache oft Kronen/Brücken auf hemisezierten Pfeilern; Erweiterung des Behandlungsrepertoires; Mache wenig Pa-Chirurgie oder BGT |
|  |  | 1. Das Gesamtkonzept der PA Therapie mit Parostatus wurde vollständig integriert und ist zukunftsfähig |
|  |  | 1. PAR-Planungen laufen komplexer ab, Ursachen werden genauer untersucht und erfasst/behandelt |
|  |  | 1. Strukturierte PA Therapie mit UPT |
|  |  | 1. Deutlichere Integration der PA Diagnostik und Therapie ins Praxiskonzept; Strukturierteres Vorgehen |
|  |  | 1. Verbesserte PARO-Diagnostik und -Therapie |
|  |  | 1. Neue Motivation durch effektivere Paro-Therapie |
|  | Gewinn von Behandlungssicherheit  N=9 | 1. Das Gefühl, besser zu wissen, was man tut, Einbindung in ein Praxiskonzept, Grenzen erweitern, Ansprechpartner für Kollegen, Übernahme von eigenen Fortbildungen |
|  |  | 1. Mehr Kompetenz und Behandlungssicherheit in diesem Fachbereich, was sich dann natürlich auch finanziell positiv auswirkt; mehr Übersicht über Therapiemöglichkeiten und Motivation für weitere Fortbildungen undSpezialisierungen |
|  |  | 1. Ich bin überzeugender, weil kompetenter geworden und hab damit auch meine Freude und mein Selbstbewusstsein beim Arbeiten verbessert |
|  |  | 1. Praktisch nichts kann mich aus der Bahn werfen, ich habe Sicherheit gewonnen in sämtlichen Behandlungsphasen |
|  |  | 1. Sehr viel Sicherheit im ganzen Konzept von Diagnose bis Therapie, mehr Zutrauen in komplexe Chirurgie, guten fachlichen Überblick über den derzeitigen wissenschaftlichen Stand |
|  |  | 1. Selbstbewussteres Auftreten |
|  |  | 1. Selbstsichereres Auftreten |
|  |  | 1. Viel größere Sicherheit in der Behandlung |
|  |  | 1. Ich behandle den Patienten deutlich sicherer, da das Studium den Blickwinkel verändert hat. Paro ist das wichtigste Fach in der Zahnheilkunde. Mit dem Wissen lassen sich viele Probleme lösen oder vermeiden. |
|  |  |  |

Tab. S4: Survey Results from March 2021 – Graduates’ Perceived Positive and Negative Changes in Their Treatment Approaches Following Participation in the Master’s Program in Periodontology and Implant Therapy at the University of Freiburg

## **Masterthesis**

| Main Category | Subcategory | Title of the Masterthesis |
| --- | --- | --- |
| Therapie  N=62 | Prophylaxe und Mundhygiene  N=15 | Elektrische Zahnbürsten im Vergleich zu Handzahnbürsten. Eine Befragung unter Patienten in einer Zahnarztpraxis. |
|  |  | Mundhygieneinstruktion: Wann macht der Patient was der Zahnarzt will? |
|  |  | Effektivität und Effizienz der Interdentalraum-Reinigung – eine vergleichende Bilanz zwischen konventionellen Hilfsmitteln und Irrigatoren |
|  |  | Effektivität unterschiedlicher Mundhygienetechniken. Literaturübersicht und Fragebogenerhebung. |
|  |  | PZR, weiße Zähne, und gesunde Gingiva? Welche Methoden sind ohne Schäden empfehlenswert? |
|  |  | Die Anwendung von Pulverstrahl-Systemen im Rahmen der systematischen Parodontitistherapie – Literaturübersicht und Dokumentation von Patientenfällen |
|  |  | Langzeiterfolge durch prophylaktische Maßnahmen nach Parodontaltherapie. Eine systematische Übersicht der Studien |
|  |  | Quellen zahnmedizinisch-prophylaktischen Wissens bei Patienten einer Praxis im Vergleich zu einer Bevölkerungsstichprobe. Eine fragebogengestützte Analyse |
|  |  | Übersichtsarbeit zur Pulverstrahltechnik in der Parodontologie |
|  |  | Stellenwert und Applikationsformen antimikrobieller Spülmittel in der sekundären und tertiären PA-Prävention, eine systematische Übersicht |
|  |  | Die Effektivität von Vakzinen und Autovakzinen in der Parodontitistherapie |
|  |  | Ölspülungen in der Parodontologie |
|  |  | Ultraschallanwendungen in der Parodontologie – Literaturübersicht und klinische Beispiele |
|  |  | Methoden und Anwendung der unterstützenden parodontalen Therapie und die Langzeiterfolge |
|  |  | Recall Compliance in der Parodontologie. Eine systematische Übersicht |
|  | Antobiotika und antimikrobielle Therapie  N=13 | Klinische Effektivität und Nebenwirkungen lokal eingesetzter Antibiotika bzw. Antiseptika in der Therapie der Parodontitis |
|  |  | Die Rolle von Metronidazol in der Parodontaltherapie |
|  |  | Vergleich Azithromycin & Metronidazol + Amoxicillin in der Parodontitistherapie. Eine Literaturübersicht |
|  |  | Antibiotikaresistenzen in der Parodontologie |
|  |  | Doxycyclin als lokales Antibiotikum in der Parodontitistherapie – eine Literaturübersicht |
|  |  | Der Winkelhoff-Cocktail in der Parodontologie – Indikationen und Grenzen |
|  |  | Adjuvante Antibiotikagabe und Photodynamische Therapie – Eine Literaturübersicht und klinische Fallbeispiele |
|  |  | Effektivität der photodynamischen Therapie mit einer LED- Lichtquelle (LED- PDT) im Vergleich zur Wirksamkeit verschiedener antiseptischer Spüllösungen an parodontalpathogenen Keimen im planktonen Modell |
|  |  | Nanomaterialien in der Parodontologie und orales Biofilmmanagement. Literaturübersicht |
|  |  | Lokale antimikrobielle Substanzen mit verzögerter Wirkstofffreisetzung in der konservativen Parodontal- und Erhaltungstherapie |
|  |  | Evidenzbasierte Wirkung von Mundspülmitteln auf orale Biofilme und parodontale Entzündungen |
|  |  | Einfluss von CHX-Applikation mittels Munddusche auf Plaqueakkumulationen (Literaturstudie) |
|  |  | Die Bedeutung von Chlorhexidin in der PA-Therapie |
|  | Parodontalchirurgie  N=8 | Sind Membranen bei infraalveolären Defekten im Vergleich zu Schmelzmatrixproteinen noch up-to-date? Stand der Wissenschaft in der Parodontologie mit Empfehlungen für den Praktiker |
|  |  | Übersichtsarbeit zur resektiven PAR Chirurgie (Indikationen, Techniken, Ergebnisse) |
|  |  | Aktuelle Schnittführungen bei minimalinvasiven/mikrochirurgischen Lappenoperationstechniken. |
|  |  | Sind freie Schleimhauttransplantate noch aktu­ell? |
|  |  | Komplikationen bei der operativen Bindegewebsentnahme aus dem Gaumen im Rahmen der plastisch-ästhetischen Parodontalchirurgie |
|  |  | Tunnelierung bei Molaren – eine Literaturübersicht |
|  |  | Therapie furkationsbefallener Molaren: Wurzelresektion versus Einzelimplantat. Eine systematische Literaturübersicht. |
|  |  | Evaluation of root coverage methods in the microsurgery (in Arbeit) |
|  | Ernährung  N=5 | Der adjunktive Einsatz von Omega-3-Fettsäuren in der Parodontitistherapie – eine Literaturübersicht sowie begleitende Case Reports |
|  |  | Probiotika |
|  |  | Klinische Effekte der adjunktiven Gabe von Vitamin C zur Parodontitistherapie – eine systematische Literaturübersicht |
|  |  | Aktueller Stand der Anwendung von Probiotika in der Parodontologie - Literaturübersicht und Praxisanwendung. |
|  |  | Der adjunktive Einsatz von Mineralstoffen in der nicht-chirurgischen Parodontitistherapie - eine systematische Literaturübersicht. |
|  | Laser  N=4 | Kürette vs. Laser: Profitiert der Patient von neu entwickelten Instrumenten? |
|  |  | Diodenlaser in der Parodontologie - Keimreduktion als Adjuvans zur konventionellen Therapie (nie abgeschlossen) |
|  |  | Antimikrobielle photodynamische Therapie (aPDT) in der Parodontologie – Aktueller Wissensstand und eigene klinische Erfahrungen |
|  |  | Die antimikrobielle photodynamische Therapie (aPDT). Review und randomisierte kontrollierte klinische Studie |
|  | Therapiefindung  N=3 | Zahnärztliche Entscheidungsfindung bei der parodontalen Erhaltungstherapie |
|  |  | Indikationen für Entscheidungen zwischen Therapieverfahren in der Parodontitistherapie. |
|  |  | Aggressive Parodontitis - Möglichkeiten und Grenzen der Therapie in der Parodontologie und den anderen Gebieten der Zahnheilkunde – Literaturarbeit |
|  | Gingivopathien  N=3 | Therapieempfehlungen chronisch-rezidivierender Aphthen. Eine Literaturübersicht |
|  |  | Behebung von Papillendefekten: Methoden und Erfolgschan­cen. |
|  |  | Therapie der Zahnhalsüberempfindlichkeit - eine Literaturübersicht. |
|  | Analgesie  N=2 | Intraligamentäre Injektion - empfehlenswert oder schädlich? |
|  |  | Schmerzen bei der nicht-chirurgischen Parodontaltherapie. Eine Literaturübersicht und Fragebogenerhebung in der eigenen Praxis. |
|  | Therapieforschung  N=2 | Was ist eine erfolgreiche Parodontalbehandlung aus Sicht des Patienten? Ergebnisse einer standardisierten Umfrage mit dem Oral Health Impact Profile (OHIP-G14) |
|  |  | Vergleich des Einsatzes von Simulationspati­enten zwischen Studierenden und Postgradu­ierten |
|  | Implantattherapie  N=2 | Aktuelle Therapieempfehlungen zur Periimplantitis – eine Literaturübersicht anhand von Reviews der letzten 5 Jahre |
|  |  | Querschnittstudie zu Langzeitergebnissen periimplantärer Weichgewebe bei implantatgestützten Doppelkronenversorgungen |
|  | Orthodontie  N=2 | Möglichkeiten der kieferorthopädischen Behandlung nach Parodontaltherapie. |
|  |  | Regeneration und Erhalt parodontaler Gewebestrukturen durch orthodontische Extrusionstechniken – eine Literaturübersicht unterstützt durch eigene klinische Fallanalysen |
|  | Digitalisierung  N=3 | lmplementation und Evaluation eines digitalen Verfahrens zum Vergleich der parodontalen Behandlungsergebnisse in niedergelassenen zahnärztlichen Praxen. |
|  |  | Eignung verschiedener Software Produkte für die Archivierung zahnärztlicher Aufnahmen |
|  |  | Digitalisierung in der Parodontologie (in Arbeit) |
| Ätiologie  N=24 | Wechselwirkungen mit systemischen Erkrankungen  N=8 | Zusammenhang von Parodontitis und Herzerkrankungen – Eine Literaturübersicht sowie begleitende Case Reports. |
|  |  | Parodontitis und Herz-Kreislauf-Erkrankungen |
|  |  | Parodontitis und Candida |
|  |  | Parodontitis und Arthritis – gibt es Zusammenhänge? |
|  |  | Parodontitis und Diabetes Mellitus – gibt es einen Zusammenhang? (in Arbeit, wird voraussichtlich nicht abgeschlossen) |
|  |  | Parodontopathien bei Papillon-Lefèvre Syndrom und Psoriasis: Ursache und Behandlung. Eine Literaturrecherche. |
|  |  | Parodontologische Behandlung von Patienten mit Bisphosphonattherapie |
|  |  | Parodontale Befunde bei Einnahme von Ca-Kanal-Blockern |
|  | Wechselwirkungen  Prothetik  N=7 | Einfluss des perioprothetischen Paradigmen­wechsels auf die parodontale Gesundheit: Veneers vs. Kronen |
|  |  | Die Bioadhäsionsprozesse auf Implantatoberflächen und Zahnhartsubstanzen - ein Vergleich. |
|  |  | Periimplantitis – eine Literaturübersicht |
|  |  | Restaurationsmaterialien und parodontale Gesundheit – Literaturarbeit |
|  |  | Zum Zusammenhang zwischen okklusaler (Über-)Belastung und Parodontopathien. Eine historische und aktuelle Analyse (auf Grundlage der Fachliteratur) |
|  |  | Bedeutung der biologischen Breite für die parodontale Gesundheit. |
|  |  | Bedeutung der biologischen Breite |
|  | Einfluss des Rauchen  N=3 | Initiale Einzelzahn-Prognose und Langzeiterhalt bei Rauchern und Nichtrauchern |
|  |  | Einfluss des Rauchens auf die parodontale Therapie |
|  |  | Rauchgewohnheiten und Raucherberatung in Zahnarztpraxen. Eine Erhebung. |
|  | Mikrobiologie  N=2 | The impact of TGF-beta 1 , BMP 4, BMP 2 in the osteogenetic differentiation of human periodontal fibroblasts and gingival fibroblasts |
|  |  | Einfluss der Kultivierdauer auf die Genexpressionsmuster von humanen Pulpastammzellen. |
|  | Gingivopathien  N=1 | Prävalenz und Ätiologie gingivaler Rezessionen – Literaturübersicht |
|  | Anatomische Wechselwirkungen  N=1 | Parodontale Probleme bei verlagerten Weisheitszähnen (in Arbeit, wird voraussichtlich nicht abgeschlossen) |
|  | Wechselwirkungen Endodontologie  N=1 | Marktote Zähne und Pa-therapie. Gibt es methodische Unterschiede zu vitalen Zähnen? |
|  | Neonatologie  N=1 | Frühgeburten und Untergewichtigkeit bei Neugeborenen durch Parodontitis bedingt? |
| Diagnostik  N=5 | Digitale Diagnostik  N=2 | Metrische Auswertung der gingivalen Weichteilmorphologie im Frontzahnbereich mit Hilfe von DVT-Daten und CADCAM-Technologie - klinischer Vergleich |
|  |  | Möglichkeiten der parodontalen Befundung mittels digitaler Volumentomographie |
|  | Laser  N=1 | "Laser in der parodontologischen Diagnostik und Therapie". Literaturübersicht mit einem in vivo-Studienanteil zur Diagnostik |
|  | Mikrobiologie  N=2 | Mikrobiologische Diagnostik in der Parodontologie und anderen ambulanten Disziplinen der Medizin. |
|  |  | MMP-8 Schnelltest vor und nach Initialtherapie |
| Epidemiologie  N=5 | Ethnische Einflüsse  N=3 | Charakterisierung der subgingivalen Flora (Biofilm) bei Parodontitis von unterschiedlichen Ethnien. |
|  |  | Die Prävalenz von Parodontitiden in Waldfeucht-Haaren unter Berücksichtigung verwandtschaftlicher Verhältnisse und modifizierender individueller Faktoren |
|  |  | Ernährungsbedingte Halitosis – Einfluss landestypischer Nahrungsmittel auf Halitosis. |
|  | Historischer Kontext  N=2 | Vergleichende Epidemiologie DMS I – IV für Mundhygiene, Gingivitis, Parodontitis, Zahnzahl und Wurzelkaries |
|  |  | Prävalenz der Parodontitis beim urgeschichtlichen Homo Sapiens - eine systematische Literaturübersicht |

Tab. S5: List of Master Thesis

## **Publications**

| **Publications** | **Journal Impact Factor** |
| --- | --- |
| **Flisfisch S, Woelber JP, Walther W.**  **Patient evaluations after local anesthesia with a computer-assisted method and a conventional syringe before and after reflection time: A prospective randomized controlled trial**  **Heliyon. 2021 Feb 8;7(2):e06012. doi: 10.1016/j.heliyon.2021.e06012.** | 3,776 |
| **Fuhrmann S, Kitzmann J, Isailov-Schöchlin M, Vach K, Fabry G, Schulz C, Jähne A, Ratka-Krüger P, Woelber JP**  **Can motivational interviewing for dental settings be taught online? Results of an uncontrolled interventional trial.Eur J Dent Educ.**  **2021 Jun 1 PMID: 34009671.** | 2,528 |
| **Kruse AB, Heil HK, Struß N, Fabry G, Silbernagel W, Vach K, Ratka-Krüger P, Woelber JP.**  **Working experience is not a predictor of good communication: Results from a controlled trial with simulated patients. Eur J Dent Educ**  **2020 May;24(2):177-185. doi: 10.1111/eje.12482. Epub 2019 Dec 12. PMID: 31765053.** | 2,355 |
| **Kruse AB, Kowalski CD, Leuthold S, Vach K, Ratka-Krüger P, Woelber JP.**  **What is the impact of the adjunctive use of omega-3 fatty acids in the treatment of periodontitis? A systematic review and meta-analysis.**  **Lipids Health Dis.**  **2020 May 21;19(1):100. doi: 10.1186/s12944-020-01267-x. PMID: 32438906; PMCID: PMC7240972.** | 3,876 |
| **Peikert SA, Mittelhamm F, Frisch E, Vach K, Ratka-Krüger P, Woelber JP.**  **Use of digital periodontal data to compare periodontal treatment outcomes in a practice-based research network (PBRN): a proof of concept. BMC Oral Health. 2020 Oct 28;20(1):297. doi: 10.1186/s12903-020-01284-3. PMID: 33115466; PMCID: PMC7594469.** | 2,757 |
| **Frisch E, Vach K, Ratka-Krueger P.**  **Impact of supportive implant therapy on peri-implant diseases: A retrospective 7-year study. J Clin Periodontol. 2020 Jan;47(1):101-109. doi: 10.1111/jcpe.13206. Epub 2019 Nov 6. PMID: 31599464.** | 5,24 |
| **Frisch E, Ratka-Krüger P.**  **A new technique for peri-implant recession treatment: Partially epithelialized connective tissue grafts. Description of the technique and preliminary results of a case series. Clin Implant Dent Relat Res. 2020 Jun;22(3):403-408. doi: 10.1111/cid.12897. Epub 2020 Mar 25. PMID: 32216023.** | 3,932 |
| **Mitschke J, Peikert SA, Vach K, Frisch E. Supportive Implant Therapy (SIT):**  **A Prospective 10-Year Study of Patient Compliance Rates and Impacting Factors. J Clin Med. 2020 Jun 25;9(6):1988. doi: 10.3390/jcm9061988. PMID: 32630385; PMCID: PMC7357043.** | 4.242 |
| **Frisch E, Wild V, Ratka-Krüger P, Vach K, Sennhenn-Kirchner S.**  **Long-term results of implants and implant-supported prostheses under systematic supportive implant therapy: A retrospective 25-year study. Clin Implant Dent Relat Res. 2020 Dec;22(6):689-696. doi: 10.1111/cid.12944. Epub 2020 Sep 23. PMID: 32969180.** | 3,932 |
| **Strenzke R, Ratka-Krüger P, Frisch E.**  **Therapy for Peri-Implantitis: Significant Radiographic Bone Fill After Keratinized Mucosa Augmentation Surgery With Supportive Implant Therapy: A Novel Approach. J Oral Implantol. 2021 Dec 1;47(6):530-534. doi: 10.1563/aaid-joi-D-20-00197. PMID: 33027806.** | 1,779 |
| **Peikert SA, Spurzem W, Vach K, Frisch E, Ratka-Krüger P, Woelber JP.**  **Association of non-surgical periodontal therapy on patients' oral health-related quality of life-A multi-centre cohort study. J Clin Periodontol. 2019 May;46(5):529-538. doi: 10.1111/jcpe.13093. Epub 2019 Apr 22. PMID: 30825387.** | 5,241 |
| **Woelber JP, Fleiner J, Rau J, Ratka-Krüger P, Hannig C.**  **Accuracy and Usefulness of CBCT in Periodontology: A Systematic Review of the Literature. Int J Periodontics Restorative Dent. 2018 Mar/Apr;38(2):289-297. doi: 10.11607/prd.2751. PMID: 29447324.** | 1,228 |
| **Ratka-Krüger P, Wölber JP, Blank J, Holst K, Hörmeyer I, Vögele E.**  **MasterOnline Periodontology and Implant Therapy-revisited after seven years: A case study of the structures and outcomes in a blended learning CPD. Eur J Dent Educ. 2018 Feb;22(1):e7-e13. doi: 10.1111/eje.12249. Epub 2016 Dec 20. PMID: 27995723.** | 1,531 |
| **Woelber JP, Ratka-Krueger P, Vach K, Frisch E. Decementation Rates and the Peri-Implant Tissue Status of Implant-Supported Fixed Restorations Retained via Zinc Oxide Cement: A Retrospective 10-23-Year Study. Clin Implant Dent Relat Res. 2016 Oct;18(5):917-925. doi: 10.1111/cid.12372. Epub 2015 Aug 12. PMID: 26265426.** | 2,939 |
| **Frisch E, Ratka-Krüger P, Weigl P, Woelber J.**  **Extraoral Cementation Technique to Minimize Cement-Associated Peri-implant Marginal Bone Loss: Can a Thin Layer of Zinc Oxide Cement Provide Sufficient Retention? Int J Prosthodont. 2016 Jul-Aug;29(4):360-2. doi: 10.11607/ijp.4599. PMID: 27479343.** | 1,386 |
| **Frisch E, Ziebolz D, Ratka-Krüger P, Rinke S.**  **Double crown-retained maxillary overdentures: 5-year follow-up. Clin Implant Dent Relat Res. 2015 Feb;17(1):22-31. doi: 10.1111/cid.12087. Epub 2013 May 16. PMID: 23679159.** | 4,152 |
| **Frisch E, Ratka-Krüger P, Ziebolz D.**  **A New Technique for Increasing Keratinized Tissue Around Dental Implants: The Partially Epithelialized Free Connective Tissue Graft. Retrospective Analysis of a Case Series. J Oral Implantol. 2015 Aug;41(4):467-72. doi: 10.1563/AAID-JOI-D-13-00006. Epub 2013 Jul 8. PMID: 23834663.** | 1,432 |
| **Frisch E, Ziebolz D, Vach K, Ratka-Krüger P.**  **The effect of keratinized mucosa width on peri-implant outcome under supportive postimplant therapy. Clin Implant Dent Relat Res. 2015 Jan;17 Suppl 1:e236-44. doi: 10.1111/cid.12187. Epub 2013 Dec 16. PMID: 24341796.** | 4,152 |
| **Frisch E, Ratka-Krüger P, Wenz HJ.**  **Unsplinted implants and teeth supporting maxillary removable partial dentures retained by telescopic crowns: a retrospective study with >6 years of follow-up. Clin Oral Implants Res. 2015 Sep;26(9):1091-7. doi: 10.1111/clr.12407. Epub 2014 Apr 16. PMID: 24734956.** | 3,464 |
| **Frisch E, Ratka-Krüger P, Ziebolz D.**  **Increasing the Width of Keratinized Mucosa in Maxillary Implant Areas Using a Split Palatal Bridge Flap: Surgical Technique and 1-Year Follow-Up. J Oral Implantol. 2015 Oct;41(5):e195-201. doi: 10.1563/aaid-joi-D-14-00025. Epub 2014 Jun 19. PMID: 24946207.** | 1,432 |
| **Rinke S, Ziebolz D, Ratka-Krüger P, Frisch E.**  **Clinical Outcome of Double Crown-Retained Mandibular Removable Dentures Supported by a Combination of Residual Teeth and Strategic Implants. J Prosthodont. 2015 Jul;24(5):358-65. doi: 10.1111/jopr.12214. Epub 2014 Sep 14. PMID: 25220099.** | 1,693 |
| **Frisch E, Ratka-Krüger P, Weigl P, Woelber J.**  **Minimizing excess cement in implant-supported fixed restorations using an extraoral replica technique: a prospective 1-year study. Int J Oral Maxillofac Implants. 2015 Nov-Dec;30(6):1355-61. doi: 10.11607/jomi.3967. PMID: 26574860.** | 1,487 |
| **Frisch E, Ziebolz D, Ratka-Krüger P, Rinke S.**  **A new technique for retaining double crowns on implants via custom-positioned vertical screws. Int J Prosthodont. 2014 Nov-Dec;27(6):577-8. doi: 10.11607/ijp.3882. PMID: 25390875.** | 1,464 |
| **Frisch E, Ziebolz D, Vach K, Ratka-Krüger P.**  **Supportive post-implant therapy: patient compliance rates and impacting factors: 3-year follow-up. J Clin Periodontol. 2014 Oct;41(10):1007-14. doi: 10.1111/jcpe.12298. Epub 2014 Sep 12. PMID: 25138992.** | 4,010 |

Tab. S6: List of publications and Journal Impact factors

| **Hauptkategorie** | **Subkategorie** | **Titel der Publikation** |
| --- | --- | --- |
| Analgesie  n=1 | Digitalisierung  N=1 | Patient evaluations after local anesthesia with a computer-assisted method and a conventional syringe before and after reflection time: A prospective randomized controlled trial |
| Parodontologie  n=16 | Ernährung  n=1 | What is the impact of the adjunctive use of omega-3 fatty acids in the treatment of periodontitis? A systematic review and meta-analysis |
|  | Fortbildungsmaßnahmen  n=1 | MasterOnline Periodontology and Implant Therapy-revisited after seven years: A case study of the structures and outcomes in a blended learning CPD |
|  | Implantattherapie  n=9 | A New Technique for Increasing Keratinized Tissue Around Dental Implants: The Partially Epithelialized Free Connective Tissue Graft. Retrospective Analysis of a Case Series |
|  |  | Impact of supportive implant therapy on peri-implant diseases: A retrospective 7-year study |
|  |  | Increasing the Width of Keratinized Mucosa in Maxillary Implant Areas Using a Split Palatal Bridge Flap: Surgical Technique and 1-Year Follow-Up |
|  |  | Long-term results of implants and implant-supported prostheses under systematic supportive implant therapy: A retrospective 25-year study |
|  |  | Supportive Implant Therapy (SIT): A Prospective 10-Year Study of Patient Compliance Rates and Impacting Factors |
|  |  | Supportive post-implant therapy: patient compliance rates and impacting factors: 3-year follow-up |
|  |  | The effect of keratinized mucosa width on peri-implant outcome under supportive postimplant therapy |
|  |  | Therapy for peri-implantitis: Significant radiographic bone fill after keratinized mucosa augmentation surgery with supportive implant therapy (SIT): A novel approach |
|  |  | A new technique for peri-implant recession treatment: Partially epithelialized connective tissue grafts. Description of the technique and preliminary results of a case series |
|  | Sprechende Therapie  N=2 | Can motivational interviewing for dental settings be taught online? Results of an uncontrolled interventional trial |
|  |  | Working experience is not a predictor of good communication: Results from a controlled trial with simulated patients |
|  | Therapieforschung  N=3 | Accuracy and Usefulness of CBCT in Periodontology: A Systematic Review of the Literature |
|  |  | Association of non-surgical periodontal therapy on patients' oral health-related quality of life-A multi-centre cohort study |
|  |  | Use of digital periodontal data to compare periodontal treatment outcomes in a practice-based research network (PBRN): a proof of concept |
| Prothetik  N=7 | Herausnehmbarer ZE  N=3 | Clinical Outcome of Double Crown-Retained Mandibular Removable Dentures Supported by a Combination of Residual Teeth and Strategic Implants |
|  |  | Double crown-retained maxillary overdentures: 5-year follow-up |
|  |  | Unsplinted implants and teeth supporting maxillary removable partial dentures retained by telescopic crowns: a retrospective study with >6 years of follow-up |
|  | Retentive Maßnahmen  N=4 | N=3A new technique for retaining double crowns on implants via custom-positioned vertical screws |
|  |  | Extraoral Cementation Technique to Minimize Cement-Associated Peri-implant Marginal Bone Loss: Can a Thin Layer of Zinc Oxide Cement Provide Sufficient Retention? |
|  |  | Minimizing excess cement in implant-supported fixed restorations using an extraoral replica technique: a prospective 1-year study |
|  |  | Decementation Rates and the Peri-Implant Tissue Status of Implant-Supported Fixed Restorations Retained via Zinc Oxide Cement: A Retrospective 10-23-Year Study |

Tab. S7: Topics of publications

| Postleitzahl | Stadt | Land |
| --- | --- | --- |
| A-1010 | Wien | A |
| A-6850 | Dornbirn | A |
| A-6900 | Bregenz | A |
| 4051 | Basel | CH |
| 6206 | Neuenkirch | CH |
| 8011 | Zürich | CH |
| 8620 | Wetzikon | CH |
| 8645 | Jona | CH |
| 8952 | Schlieren | CH |
| 9008 | St. Gallen | CH |
| 8050 | Zürich | CH |
| 10021 | Berlin | D |
| 6270 | Merseburg | D |
| 10437 | Berlin | D |
| 10585 | Berlin | D |
| 10711 | Berlin | D |
| 10711 | Berlin | D |
| 12165 | Berlin | D |
| 14057 | Berlin | D |
| 14057 | Berlin | D |
| 19073 | Wittenförden | D |
| 21465 | Reinbek | D |
| 22087 | Hamburg | D |
| 22145 | Hamburg | D |
| 22399 | Hamburg | D |
| 22453 | Hamburg | D |
| 22587 | Hamburg | D |
| 22765 | Hamburg | D |
| 23552 | Lübeck | D |
| 24107 | Kiel | D |
| 26121 | Oldenburg | D |
| 29358 | Eicklingen | D |
| 30173 | Hannover | D |
| 34369 | Hofgeismar | D |
| 49838 | Lengerich | D |
| 50937 | Köln | D |
| 52538 | Selfkant | D |
| 52538 | Selfkant | D |
| 55118 | Mainz | D |
| 55131 | Mainz | D |
| 60431 | Frankfurt a.M. | D |
| 60529 | Frankfurt | D |
| 64625 | Bensheim | D |
| 65428 | Rüsselsheim | D |
| 66957 | Vinningen | D |
| 67433 | Neustadt an der Weinstraße | D |
| 70174 | Stuttgart | D |
| 70199 | Stuttgart | D |
| 72072 | Tübingen | D |
| 72074 | Tübingen | D |
| 72076 | Tübingen | D |
| 72108 | Rottenburg am Neckar | D |
| 72581 | Dettingen | D |
| 74074 | Heilbronn | D |
| 74074 | Heilbronn | D |
| 75015 | Bretten | D |
| 76530 | Baden-Baden | D |
| 76870 | Kandel | D |
| 77652 | Offenburg | D |
| 77716 | Haslach | D |
| 77855 | Achern | D |
| 78315 | Radolfzell | D |
| 78315 | Radolfzell | D |
| 78464 | Konstanz | D |
| 79102 | Freiburg | D |
| 79102 | Freiburg | D |
| 79189 | Bad Krozingen | D |
| 79199 | Kirchzarten | D |
| 79206 | Breisach | D |
| 79249 | Merzhausen | D |
| 79268 | Bötzingen | D |
| 79822 | Titisee-Neustadt | D |
| 82110 | Germering | D |
| 82152 | Krailing | D |
| 83022 | Rosenheim | D |
| 83071 | Stephanskirchen | D |
| 83370 | Roitham/Seeon | D |
| 85072 | Eichstätt | D |
| 85435 | Erding | D |
| 86609 | Donauwörth | D |
| 86825 | Bad Wörishofen | D |
| 86825 | Bad Wörishofen | D |
| 88073 | Ulm | D |
| 88239 | Wangen | D |
| 89073 | Ulm | D |
| 90762 | Fürth | D |
| 92637 | Weiden | D |
| 93458 | Eschlkam | D |
| 93458 | Eschlkam | D |
| 97980 | Bad Mergentheim | D |
| 06333 | Hettstedt | D |
| 6108 | Halle-Saale | D |
| 15732 | Eichwalde | D |
| 46446 | Emmerich | D |
| 60389 | Frankfurt am Main | D |
| 65193 | Wiesbaden | D |
| 72488 | Sigmaringen | D |
| 73240 | Wendlingen | D |
| 79859 | Schluchsee | D |
| 80809 | München | D |
| 86473 | Ziemetshausen | D |
|  | Blackrock, Co. Dublin | IRL |
| 6291 | Hn Vaals | NL |
| 6511 | Nijmegen | NL |
| 5537 | Haugesund | NO |
| 4550 | Farsund | NOR |

Tab. S8: Locations of alumni
